# Dual checkpoint blockade of CD47 and LILRB1 enhances CD20 antibody-dependent phagocytosis of lymphoma cells by macrophages

**DOI:** 10.3389/fimmu.2022.929339

**Published:** 2022-10-27

**Authors:** Tobias Zeller, Sebastian Lutz, Ira A. Münnich, Roland Windisch, Patricia Hilger, Tobias Herold, Natyra Tahiri, Jan C. Banck, Oliver Weigert, Andreas Moosmann, Michael von Bergwelt-Baildon, Cindy Flamann, Heiko Bruns, Christian Wichmann, Niklas Baumann, Thomas Valerius, Denis M. Schewe, Matthias Peipp, Thies Rösner, Andreas Humpe, Christian Kellner

**Affiliations:** ^1^ Division of Transfusion Medicine, Cell Therapeutics and Haemostaseology, University Hospital, LMU Munich, Munich, Germany; ^2^ Department of Medicine III, University Hospital, LMU Munich, Munich, Germany; ^3^ German Cancer Consortium (DKTK), Partner Site Munich, Munich, Germany; ^4^ German Cancer Research Center (DKFZ), Heidelberg, Germany; ^5^ DZIF – German Center for Infection Research, Munich, Germany; ^6^ Helmholtz Zentrum München, Munich, Germany; ^7^ Department of Internal Medicine 5, University Hospital Erlangen, Erlangen, Germany; ^8^ Division of Stem Cell Transplantation and Immunotherapy, Department of Internal Medicine II, Christian Albrechts University and University Hospital Schleswig-Holstein, Kiel, Germany; ^9^ Department of Pediatrics, Otto-von-Guericke University Magdeburg, Magdeburg, Germany; ^10^ Division of Antibody-Based Immunotherapy, Department of Internal Medicine II, Christian Albrechts University and University Hospital Schleswig-Holstein, Kiel, Germany

**Keywords:** antibody therapy, macrophages, phagocytosis, CD20, CD47, LILRB1 (ILT2), innate immune checkpoint blockade, lymphoma

## Abstract

Antibody-dependent cellular phagocytosis (ADCP) by macrophages, an important effector function of tumor targeting antibodies, is hampered by ‘Don´t Eat Me!’ signals such as CD47 expressed by cancer cells. Yet, human leukocyte antigen (HLA) class I expression may also impair ADCP by engaging leukocyte immunoglobulin-like receptor subfamily B (LILRB) member 1 (LILRB1) or LILRB2. Analysis of different lymphoma cell lines revealed that the ratio of CD20 to HLA class I cell surface molecules determined the sensitivity to ADCP by the combination of rituximab and an Fc-silent variant of the CD47 antibody magrolimab (CD47-IgGσ). To boost ADCP, Fc-silent antibodies against LILRB1 and LILRB2 were generated (LILRB1-IgGσ and LILRB2-IgGσ, respectively). While LILRB2-IgGσ was not effective, LILRB1-IgGσ significantly enhanced ADCP of lymphoma cell lines when combined with both rituximab and CD47-IgGσ. LILRB1-IgGσ promoted serial engulfment of lymphoma cells and potentiated ADCP by non-polarized M0 as well as polarized M1 and M2 macrophages, but required CD47 co-blockade and the presence of the CD20 antibody. Importantly, complementing rituximab and CD47-IgGσ, LILRB1-IgGσ increased ADCP of chronic lymphocytic leukemia (CLL) or lymphoma cells isolated from patients. Thus, dual checkpoint blockade of CD47 and LILRB1 may be promising to improve antibody therapy of CLL and lymphomas through enhancing ADCP by macrophages.

## Introduction

Therapeutic antibodies are well established in the treatment of cancer (1). In B-cell Non-Hodgkin lymphomas (B-NHL), tumor targeting CD20 antibodies such as rituximab and obinutuzumab have considerably improved the patient outcome (2). However, individual patients fail to respond and relapsed or refractory disease remains challenging. Progress was made by the approval of chimeric antigen receptor T cell therapies, tailor-made fragment crystallizable (Fc)-engineered antibodies, bispecific antibodies and T cell immune checkpoint inhibitors (1, 3–6). Besides, antibodies targeting checkpoints in innate immune cells such as natural killer (NK) cells or myeloid cells have gained increasing attention (7). In particular, they may hold the potential to boost key functions of typical tumor targeting antibodies such as antibody dependent cell-mediated cytotoxicity (ADCC) or phagocytosis (ADCP) (1, 7, 8).

Several studies highlighted that macrophages, which in humans express the activating immunoglobulin γ Fc region receptors (FcγR) FcγRI, FcγRIIA and FcγRIIIA as well as inhibitory FcγRIIB, represent major effector cells for rituximab and other therapeutic antibodies (2, 9, 10). According to their functional polarization, macrophages were roughly categorized into classically activated, pro-inflammatory M1 macrophages and alternatively activated, anti-inflammatory M2 macrophages. This classification was later refined by grouping the latter into M2a, M2b, M2c and M2d subtypes to take account of distinct stimuli and diverse functional properties (11, 12). However, macrophages exert a high level of plasticity and M1 and the different M2 states represent the extreme ends in a broad spectrum of different functional states. Also, subsets with intermediate phenotypes exist (12). Macrophages are among the most frequent normal cells in the tumor microenvironment (9). High numbers of tumor-associated macrophages (TAM), which often become tumor-edited into a pro-tumorigenic M2-like state, correlated with tumor progression and poor prognosis (13–16). Yet, during antibody therapy, macrophages may contribute to tumor eradication by eliminating malignant cells directly by ADCC and ADCP or by promoting adaptive immune responses by presenting tumor antigens to T cells (8, 10). Thus, studies in B-NHL patients receiving rituximab suggested that a high content of TAM correlated with improved survival and that rituximab therapy abrogated the correlation between high numbers of TAM and poor prognosis (15, 17, 18). The anti-tumoral functions of macrophages during antibody therapy may be further promoted by certain chemotherapeutic agents, as demonstrated for example for cyclophosphamide in a murine model of B cell leukemia (19).

In regard of the important role of macrophages, strategies were developed to improve their recruitment and ADCP function. These approaches include Fc engineering to enhance the antibody`s affinity to activating FcγR (20, 21) as well as the blockade of inhibitory checkpoints that interfere with FcγR signaling (10). Signal-regulatory protein (SIRP) α is one of the best characterized myeloid inhibitory receptors. SIRPα recognizes the ubiquitously expressed ‘Don`t Eat Me!’ signal molecule CD47, and antibody blockade of either CD47 or SIRPα strongly enhanced ADCP by macrophages (22, 23). For example, the combination of rituximab with a CD47 antibody increased ADCP of lymphoma cells by macrophages *in vitro* and improved the therapeutic efficacy in xenograft models of B-NHL (24). Moreover, accompanying CD38 antibody therapy with CD47 checkpoint blockade demonstrated efficacy in patient-derived T cell acute lymphoblastic leukemia xenograft models (25). At present, monoclonal CD47 antibodies, anti-SIRPα antibodies and SIRPα-Fc fusion proteins, as well as small molecule inhibitors are in different stages of pre-clinical or clinical development (23, 26–30). Promising results were obtained with the combination of rituximab plus the CD47 antibody hu5F9-G4 (magrolimab) in a clinical phase Ib study in B-NHL patients (28). Although the IgG4 antibody magrolimab was well tolerated by the patients, safety of CD47 targeting remains a serious concern due to the ubiquitous CD47 expression by normal cells, which may cause on-target toxicity. In particular, CD47 expressing red blood cells (RBC), which display cell surface “Eat Me!” signals and downregulate CD47 during their lifespan, are vulnerable to CD47 antibody therapy. This may result in enhanced RBC clearance during CD47 antibody therapy, even when antibody formats with diminished Fc-mediated functions such as IgG4 isotypes are employed (10, 28).

Leukocyte immunoglobulin-like receptor (LILR) B 1 may represent another target for immune checkpoint blockade in monocytes and macrophages (10, 31). LILRB1 and other members of the inhibitory subfamily B of LILR share structural similarities with human killer cell immunoglobulin-like receptors (KIR) and are characterized by cytoplasmic immunoreceptor tyrosine-based inhibition motifs (ITIM) (32). In addition to several pathogen-derived ligands, LILRB1 binds classical human leukocyte antigen (HLA) class I as well as non-classical HLA-G and HLA-F molecules by interaction with the α3 domain and β2-microglobulin (33–35). Recently, it has been shown that antibody blockade of either LILRB1 or HLA-class I promotes phagocytosis of solid tumor cells, that genetic ablation of cell surface expression of HLA class I and CD47 augmented ADCP by anti-EpCAM or anti-EGFR antibodies and that MHC class I expression confers protection from macrophages in a murine tumor model (36). In addition, macrophages express LILRB2, another inhibitory receptor for HLA class I molecules that interferes with FcγR signaling (37, 38). Interestingly, LILRB2 antagonism was suggested to reprogram tumor-associated myeloid cells, to enhance pro-inflammatory responses and to promote antitumor effects of T cell immune checkpoint inhibitors (39). However, whether LILRB2 directly impairs phagocytosis is currently not known (10).

Here, the impact of HLA class I expression on macrophage-mediated ADCP of lymphoma cells and the potential of antibodies blocking the cognate inhibitory receptors LILRB1 and LILRB2 was analyzed. To prevent the induction of any Fc-mediated effects by these blocking antibodies, which may cause difficulties in unraveling effects mediated by FcγR engagement or receptor blockade, Fc-silent IgGσ variants were used, in which both FcγR and complement binding was abrogated by Fc engineering (40, 41). Clinically, such Fc-silent immune checkpoint blocking antibodies may cause less on-target side effects, as they do not induce ADCC, ADCP or CDC on their own and avoid cross-linkage of antibody bound receptors by effector cells through Fc-FcγR interactions. The anti-LILRB1 antibody was found to significantly enhance ADCP when combined with CD20 and CD47 antibodies by analyzing different B-NHL cell lines and tumor cells from chronic lymphocytic leukemia (CLL) or mantle cell lymphoma (MCL) patients.

## Materials and methods

### Antibodies and reagents

The antibodies rituximab, obinutuzumab and trastuzumab (Hoffmann-La Roche AG, Basel, Switzerland) were provided by the institutional pharmacy. The allophycocyanin (APC)-labeled CD11b antibody (clone M1/70) as well as phycoerythrin (PE)-conjugated antibodies specific for CD80 (clone REA661), CD163 (clone REA812), LILRB1 (clone REA998) or LILRB2 (clone REA184) and the corresponding isotype antibody (clone REA293) were from Miltenyi Biotec (Bergisch Gladbach, Germany). The APC-conjugate of the anti-LILRB1 antibody clone GHI/75 as well as Brilliant Violet 421-conjugated CD163 (GHI/61) and Brilliant Violet 510-coupled CD15 (W6D3) antibodies were purchased from BioLegend (San Diego, CA, USA). Murine antibodies against cell surface antigens CD20 (clone S1815E), HLA-A,-B,-C (clone W6/32), HLA-G (clone 87G), SIRPα (clone 15-414), LILRB1 (clone GHI/75), CD47 (clone B6H12) and an IgG2a isotype (clone MOPC-173) were purchased from BioLegend. The anti-LILRB2 antibody (clone 287219) and an IgG1 istotype antibody (clone 11711) were obtained from R&D systems, Inc. (Minneapolis, MN, USA). Secondary PE-conjugated F(ab’)_2_ fragments of goat anti-human Fcγ region antibodies were purchased from Jackson ImmunoResearch Laboratories, Inc. (West Grove, PA, USA). Human recombinant macrophage colony stimulating factor (M-CSF), granulocyte macrophage-colony stimulating factor (GM-CSF), interferon (IFN)-γ and interleukin-10 (IL-10) were purchased from PeproTech (Cranbury, NJ, USA). Lipopolysaccharide (LPS) O127:B8 was obtained from Sigma-Aldrich (St. Louis, MO, USA).

### Cell culture

Carnaval, DG-75 and SU-DHL-4 (German Collection of Microorganisms and Cell Cultures GmbH, DSMZ, Braunschweig, Germany) cells were cultured in Roswell Park Memorial Institute 1640 medium (Thermo Fisher Scientific, Waltham, MA, USA) supplemented with 10% fetal calf serum (FCS, Thermo Fisher Scientific) and 1% penicillin (Pen)/streptomycin (Strep) solution (Lonza, Basel, Switzerland). Granta 519 (DSMZ), Chinese hamster ovary (CHO)-K1 (DSMZ) and Lenti-X™ 293T cells (Clontech, Saint-Germain-en-Laye, France) were kept in Dulbecco’s Modified Eagle’s Medium (Thermo Fisher Scientific) containing 10% FCS and 1% Pen/Strep. MEC2 cells (DSMZ) were maintained in Iscove’s Modified Dulbecco’s Medium (Thermo Fisher Scientific) supplemented with 20% FCS and 1% Pen/Strep. Cells were cultured in a humidified atmosphere at 37°C and 6% CO_2_.

### Isolation of mononuclear cells and generation of macrophages

Experiments were approved by the Ethics Committee of the faculty of medicine, LMU Munich (18-821 and 21-0816), in accordance with the Declaration of Helsinki. Blood samples were collected after receiving the donors` written informed consents. MNC were isolated from peripheral blood or leukoreduction system chambers by density gradient centrifugation using Ficoll^®^ Paque Plus (Cytiva, Marlborough, MA, USA). Monocytes were isolated by plastic adherence using monocyte attachment medium (PromoCell GmbH, Heidelberg, Germany) following the manufacturer`s protocols. To generate non-polarized M0 macrophages adherent monocytes were cultivated in X-Vivo™ 15 (Lonza) medium supplemented with 0.5% Pen/Strep and, unless otherwise indicated, M-CSF at a concentration of 50 ng/ml for 7 days. In individual experiments, M-CSF was replaced by GM-CSF (10 ng/ml). If not specified, polarized M1 macrophages were obtained by culturing cells in the presence of GM-CSF (10 ng/ml) for 6 days to drive macrophage differentiation towards an M1 phenotype, before stimulation with IFN-γ (10 ng/ml) and LPS (100 ng/ml) for additional 48 h. M2 macrophages (M2c subtype) were polarized with M-CSF (50 ng/ml) for 6 days, before IL-10 was added at a concentration of 10 ng/ml for additional 48 h. Macrophages were harvested by accutase (Thermo Fisher Scientific) treatment following the manufacturer`s recommendations.

### Isolation of bone marrow macrophages from lymphoma patients

Macrophages, defined as CD163-positive and CD15-negative cells, were isolated from cryo-preserved BM-derived samples from diffuse large B cell lymphoma (DLBCL) patients by density-gradient centrifugation and fluorescence activated cell sorting as described previously (42). Bone marrow samples from DLBCL patients with BM infiltration served as a source for lymphoma associated macrophages (LAM). The purity was greater than 95%. Each patient gave informed consent prior to surgery or bone marrow biopsy, and the institutional ethics committee approved the study (Erlangen: Ref. number 21-403-Br).

### Cloning, expression and purification of recombinant antibodies

Anti-LILRB1 and anti-LILRB2 antibodies were derived from the sequences from the antibody clones GHI/75 and 19.h1, respectively (43, 44). As a murine IgG1 antibody GHI/75 has been demonstrated to block HLA class I/β2M binding to LILRB1, thereby promoting phagocytosis of cancer cells (36). The antibody clone 19.h1 is a humanized version of antibody 19.1, which had been selected for its abilities to block interactions between HLA class I and LILRB2 and to shift macrophage polarization towards an M1 phenotype (44). For construction of the Fc-silent anti-LILRB1 antibody (LILRB1-IgGσ), DNA fragments encoding the variable light (VL) and heavy (VH) chains of antibody GHI/75 as well as the constant human κ light (LC) and IgG2σ heavy chain (HC) regions (amino acid substitutions: V234A/G237A/P238S/H268A/V309L/A330S/P331S) were synthesized *de novo* (Thermo Fisher Scientific) according to published sequences (40, 45). LC and HC sequences were cloned into vector pSecTag2/Hygro C (Thermo Fisher Scientific) using NheI/PmeI restriction sites. To generate an IgG1σ (amino acid substitutions L234A/L235A/G237A/P238S/H268A/A330S/P331S) variant (41) of the anti-LILRB2 antibody 19.h1 (LILRB2-IgGσ), VL and VH sequences were synthesized *de novo* (Thermo Fisher Scientific) conforming to published sequences (44) and ligated into vectors pSecTag2-LC (20) and pSecTag2-HC-IgG1σ (C Kellner, unpublished) as NheI/HindIII and NheI/PpuMI cassettes, respectively. For generation of antibody CD47-IgGσ, VL and VH regions of antibody hu5F9 (46) were synthesized *de novo* and cloned into vectors pSec-CD3-HC-IgG2σ (M. Peipp, unpublished) and pSecTag2-LC (20) as NheI/HindIII and NheI/PpuMI cassettes, respectively. The expression vector for the HC of an Fc-engineered version of rituximab (RTX-DE; amino acid substitutions S239D/I332E) was generated by excising rituximab VH regions from vector pSecTag2-CD20-HC (47) and ligation into vector pSecTag2-HC-DE (20) using NheI/PpuMI restriction sites. The vectors encoding rituximab LC, trastuzumab LC or an Fc-engineered HC of trastuzumab (HER2-DE; amino acid substitutions S239D/I332E) have been described previously (20, 47). The vector encoding an Fc-silent IgG2σ HC variant of trastuzumab (HER2-IgGσ) was obtained by ligation of trastuzumab VH chain sequences in vector pSec-CD3-HC-IgG2σ using NheI/PpuMI restriction sites. For expression, Lenti-X™ 293T cells were co-transfected with HC and LC expression vectors by calcium phosphate transfection with chloroquine following standard protocols. Cell culture supernatants were collected for six days. Antibodies were purified by affinity chromatography using CaptureSelect™ IgG-CH1 affinity matrix (Thermo Fisher Scientific) as described earlier (47) and dialyzed against PBS (Thermo Fisher Scientific). The IgA2 isotype variants of rituximab (RTX-IgA2) and the anti-epidermal growth factor receptor (EGFR) antibody cetuximab have been described previously and were expressed in CHO-S cells (Thermo Fisher Scientific) by transient transfection following published procedures (48–50).

### Microfluidic chip electrophoresis

Purity, integrity and concentrations of the purified antibodies were determined by microfluidic chip electrophoresis under reducing and non-reducing conditions. Four microliters of antibody preparations were analyzed using the Agilent Protein 230 Kit and the Agilent 2100 Bioanalyzer system (Agilent Technologies, Santa Clara, CA, USA) following the manufacturer’s recommendations.

### Sodium dodecyl sulfate polyacrylamide gel electrophoresis (SDS PAGE) and Western Transfer experiments

SDS-PAGE and Western Transfer experiments were performed using standard procedures as described elsewhere (47). Antibody LC and HC were detected with horseradish peroxidase (HRP)-conjugated goat anti human κ light chain (Bio-Rad Laboratories, Inc.) and goat anti-human IgG/Fc specific (Merck, Darmstadt, Germany) antibodies, respectively.

### Gel filtration

Gel filtration was performed using the ÄKTApure protein purification system (Cytiva). For analysis, 100 - 300 µg of antibodies were loaded on a Superdex™ 200 Increase 10/300 GL column (Cytiva) and analyzed at a flow speed of 0.75 ml/min using PBS as running buffer.

### Expression of cell surface antigens by transient transfection

DNA sequences encoding human full-length LILRB1 and LILRB2 proteins (UniProtKB accession numbers Q8NHL6 and Q8N423, respectively) were synthesized *de novo* (Thermo Fisher Scientific) and cloned into expression vector pcDNA 3.1 (+) (Thermo Fisher Scientific). CHO-K1 cells were transfected using Lipofectamine^®^ LTX and Plus™ Reagent (Thermo Fisher Scientific) according to the manufacturer’s recommendation. After 48 h, the transfected cells (i.e. CHO-LILRB1 and CHO-LILRB2, respectively) were used in functional analysis.

### Flow cytometry

Flow cytometry experiments were performed on a FACSCalibur™ flow cytometer (BD Biosciences, Franklin Lakes, NJ, USA) with the exception of multi-colour analysis, which were performed on a FACSCanto II cytometer (BD Biosciences). Three hundred thousand cells were washed once with 2 ml PBS containing 1% bovine serum albumin (FACS buffer). In direct immunofluorescence assays, cells were incubated with fluorescent dye-conjugated antibodies at dilutions recommended by the manufacturer in FACS buffer at 4°C for 60 min. Expression of cell surface antigens was quantified by calibrated indirect flow cytometry using Qifikit^®^ (Dako, Glostrup, Denmark) according to the manufacturer’s recommendations. Murine antibodies were applied at a concentration of 20 µg/ml in FACS buffer supplemented with 1 mg/ml pooled human immunoglobulin (Gamunex^®^ 10%; Grifols, Barcelona, Spain) to block FcγR. Binding of chimeric or humanized antibodies was analyzed by incubation of cells with antibodies at a concentration of 50 µg/ml at 4°C for 60 min. Cells were washed once with 2 ml of FACS buffer. F(ab’)_2_ fragments of goat anti-human IgG, Fcγ fragment specific antibodies were used for detection. To analyze the ability of anti-LILRB1 and anti-LILRB2 antibodies to block receptor binding by HLA class I molecules, 0.3 × 10^6^ CHO-LILRB1 or CHO-LILRB2 cells were incubated with the antibodies at a concentration of 50 µg/ml in 20 µl FACS buffer for 1 h at 4°C. Then, 3 µl of PE-conjugated MHC I Dextramer^®^ of CMV pp65 peptide (NLVPMVATV)-loaded HLA-A*0201 (Immudex, Kopenhagen, Denmark) were added. Cells were incubated for 30 min, washed with FACS buffer, and analyzed. Mean fluorescence intensity (MFI) values were normalized to the control reaction and residual binding of HLA molecules was calculated. In all experiments, appropriate scatter gates were applied to exclude debris or dead cells and 10,000 events were counted. Data were analyzed using FlowJo v10.7.2 software (Becton Dickinson).

### Analysis of ADCP by fluorescence microscopy

Twenty thousand macrophages per well were plated in M0, M1 or M2c macrophage differentiation media on 8 well µ-slides (Ibidi GmbH, Graefelfing, Germany) and incubated overnight. Lymphoma cells were labeled using carboxyfluorescein succinimidyl ester (CFSE) Cell Division Tracker Kit (BioLegend) according to the manufacturer’s recommendations. Forty thousand lymphoma cells were added to each well in a final volume of 300 µl. Antibodies were applied and cells were incubated at 37°C with 6% CO_2_ for 2 h. Non-phagocytosed target cells were removed by exchanging the supernatant by fresh medium. ADCP was determined by counting the number of phagocytosed tumor cells per individual macrophages using the fluorescence microscope Axio Observer D1 (Carl Zeiss AG, Oberkochen, Germany), unless otherwise indicated. Fifty to 100 macrophages were analyzed. The phagocytic index was calculated by the equation: Phagocytic index = (number of engulfed target cells/number of macrophages) × 100. In individual experiments, cytoplasmic membranes of macrophages and nuclei were stained with CellBrite™ Orange Cytoplasmic Membrane Labeling Dye (Biotium, Inc., Fremont, CA, USA) and NucBlue™ Live ReadyProbes™ (Thermo Fisher Scientific), respectively, according to the manufacturer’s recommendations.

The phagocytic activity of LAM was analyzed by plating purified LAM in 8-chamber slides (Thermo Fisher Scientific). LAM were co-incubated for 2 h with Cytolight Rapid Green (Sartorius, Göttingen, Germany) labeled Carnaval cells (E:T cell ratio: 1:1) in the presence of the indicated antibodies at a concentration of 1 µg/ml. The adherent cells were washed with PBS, stained with an APC-conjugated CD11b antibody, and were fixed with 4% paraformaldehyde in PBS. The slide was then overlaid with DAPI medium and covered with a glass cover slide. Slides were analyzed using a confocal microscope (LSM700, Carl Zeiss AG).

### Live cell imaging

To analyze the phagocytic uptake of target cells, 4 × 10^4^ macrophages were plated in 50 µl of M0, M1 or M2c macrophage differentiation media in 96-well cell culture plates (Greiner Bio-One GmbH, Frickenhausen, Germany) and allowed to settle at 37°C with 6% CO_2_ for at least 1 h. Antibodies were added at the indicated concentrations. Target cells were labeled with the pH-sensitive labeling dye pHrodo^®^ (Sartorius AG) at 500 ng/ml according to the manufacturer’s recommendations. Per well, 8 × 10^4^ target cells were added in 50 µl X-Vivo™ 15 medium and live cell imaging was initiated using the IncuCyte^®^ system (Sartorious AG). Four images per well were recorded every 30 minutes and red object counts per image were determined.

To analyze the depletion of target cells, CFSE-labeled target cells were co-cultured with macrophages in the presence of antibodies in 8 well µ-slides (Ibidi GmbH) as described above for ADCP analysis by fluorescence microscopy. After 2 h, the supernatant was carefully resuspended without disturbing adherent macrophages. For quantitation, 100 µl were transferred to a 96-well plate, pelleted by centrifugation and green fluorescent cells were counted using the Incucyte^®^ system by analyzing nine images per well. Relative residual numbers of target cells were determined by normalizing data to the control reaction performed in the absence of antibodies and the percentage of target cell depletion was calculated.

### Data processing and statistical analyses

Statistical and graphical analyses were performed using the GraphPad Prism 8.0.2 software (GraphPad Software, La Jolla, CA, USA). Statistically significant differences between treatment groups were assessed using two-sided Student´s t-test, one-way or two-way ANOVA and Šidàk´s, Tukey´s or Fisher´s LSD post-tests, as indicated. The correlation between the ratio of CD20 to HLA class I molecules per cell and susceptibility to ADCP was calculated using the Pearson correlation test. P-values ≤ 0.05 were considered statistically significant.

## Results

**Figure 1:**
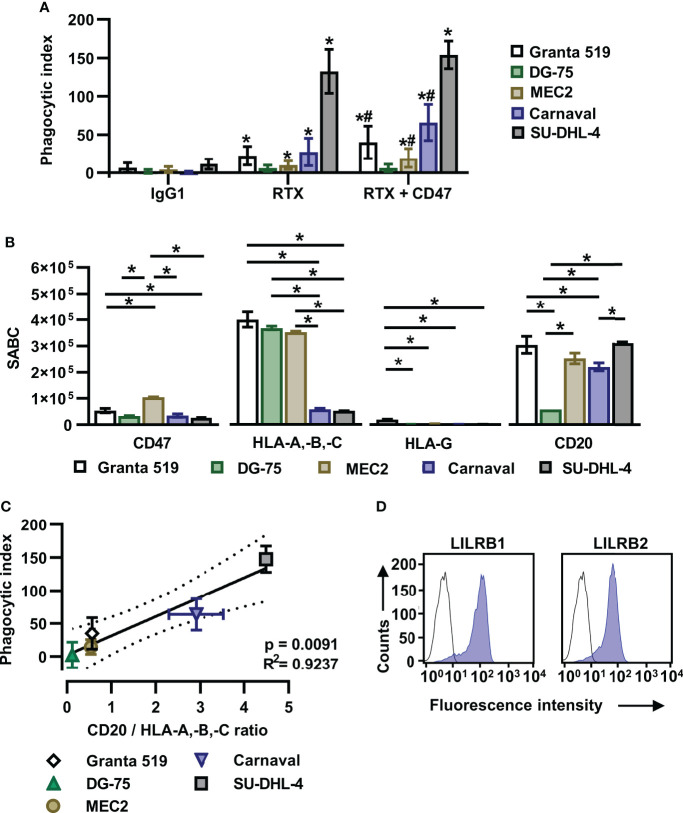
The ratio of CD20 to HLA-A,-B,-C antigen expression determines the efficacy of the combination of rituximab and a CD47 blocking antibody in initiating ADCP. **(A)** Human M0 macrophages were incubated with CFSE-labeled Granta 519 (*n* = 8), DG-75 (*n* = 6), MEC2 (*n* = 10), Carnaval (*n* = 11) or SU-DHL-4 (*n* = 4) lymphoma cells (E:T cell ratio: 1:2) in the presence of either rituximab (RTX) or a combination of RTX plus CD47-IgGσ (CD47) for 2 h. ADCP was analyzed by fluorescence microscopy and the phagocytic index was calculated. Trastuzumab (IgG1) was used as control. Antibodies were applied at 10 µg/ml. Bars represent mean values ± SD. Statistically significant differences (P ≤ 0.05) in ADCP compared to treatment with IgG1 (*) or RTX (#) are indicated (Two-way ANOVA with Tukey’s multiple comparisons test). **(B)** Lymphoma cells were stained with antibodies specific for CD20, CD47, HLA-A,-B,-C or HLA-G and specific antibody binding capacities (SABC) were determined by calibrated flow cytometry. Bars indicate mean values ± SEM (*n* = 3). Statistically significant differences between individual lymphoma cell lines are indicated (*P ≤ 0.05; one-way ANOVA with Tukey’s *post hoc* test). **(C)** For each lymphoma cell line the ratio of CD20 to HLA-A,-B,-C expression was determined and plotted against the calculated mean phagocytic index values for treatment with RTX plus CD47-IgGσ. The solid line represents the best-fit curve, dotted lines indicate the 95% confidence interval. Error bars indicate SD. **(D)** M0 macrophages were stained with murine antibodies against LILRB1 or LILRB2 (blue shaded peaks) or an isotype control antibody (black outlined peaks). Secondary FITC-conjugated goat anti-mouse immunoglobulins (DAKO) were used for detection. Cell surface expression was analyzed by flow cytometry. One representative experiment is shown (*n* = 7).

In an effort to define critical determinants of ADCP of lymphoma cells, we analyzed the sensitivity of the lymphoma cell lines Granta 519 (MCL), Carnaval (DLBCL), DG-75 (Burkitt lymphoma), MEC2 (CLL) and SU-DHL-4 (DLBCL) using human, non-polarized M0 macrophages. To induce ADCP, cells were treated with the CD20 antibody rituximab or a combination of rituximab and a variant of the CD47 antibody magrolimab with abrogated FcγR binding (referred to as CD47-IgGσ; **Supplementary Figure 1**). Each antibody was analyzed at the saturating concentration of 10 µg/ml. The analysis by fluorescence microscopy revealed that the extent of ADCP induced by rituximab or by the combination of rituximab plus CD47-IgGσ differed considerably between the cell lines. The CD47 antibody, which was unable to trigger ADCP on its own due to abrogated FcγR binding, enhanced ADCP by rituximab in Carnaval, MEC2 and Granta 519 cells significantly. Minor add-on effects from the CD47 antibody were observed with DG-75 cells, which were hardly engulfed even in the presence of both antibodies, and SU-DHL-4 cells, which were extraordinarily sensitive to rituximab-mediated ADCP (**Figure 1A**). Regarding the ‘Don´t Eat Me!’ function described for HLA class I molecules by interaction with the inhibitory receptor LILRB1, we hypothesized that their expression interfered with ADCP of lymphoma cells and contributed to the observed differences. Therefore, the cell surface expression levels of HLA class I molecules, the rituximab target antigen CD20 and CD47 were determined (**Figure 1B**). The cell lines showed diverging expression of these antigens, with high variation in the expression of classical HLA class I molecules (HLA-A,-B,-C) and CD20, while moderate differences in CD47 expression were observed. HLA-G expression was not detectable with the exception of Granta 519 cells. Although a correlation between the expression of either HLA class I or CD20 and ADCP by combination treatment with rituximab and the CD47 antibody was not found (**Supplementary Figure 2**), a correlation between ADCP and the expression ratio of CD20 to HLA class I molecules was observed (**Figure 1C**; **Supplementary Table 1**). Thus, the number of displayed binding sites for rituximab and inhibitory HLA class I molecules contributed to determining the sensitivity to ADCP.

**Figure 2:**
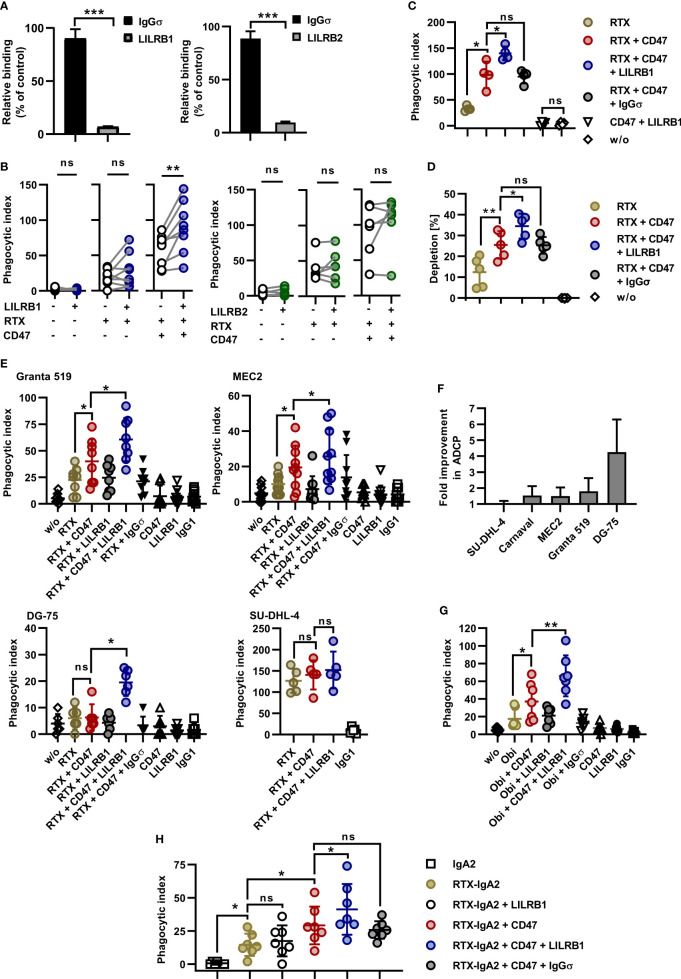
Dual checkpoint blockade of LILRB1 and CD47 enhances ADCP of lymphoma cells. **(A)** CHO-K1 cells transiently transfected with LILRB1 (*left graph*) or LILRB2 expression vectors (*right graph*) were incubated with antibodies LILRB1-IgGσ (LILRB1) and LILRB2-IgGσ (LILRB2), respectively, at a concentration of 50 µg/ml to mask LILRB receptors. Antibody HER2-IgGσ (IgGσ) was used as a control. Cells were then reacted with PE-conjugated MHC I Dextramer^®^ of NLVPMVATV-peptide-loaded HLA-A*0201 molecules. Mean fluorescence intensity was determined by flow cytometry and residual binding of HLA molecules relative to the control was calculated. Bars represent mean values ± SEM (*n* = 3; ***P ≤ 0.001; Student’s t test). **(B)** LILRB1-IgGσ (LILRB1; *left*, *n* = 8) and LILRB2-IgGσ (LILRB2; *right*, *n* = 6) were analyzed in ADCP reactions using human M0 macrophages and CFSE-labeled Carnaval cells (E:T cell ratio: 1:2). The antibodies (concentration: 10 µg/ml) were tested alone, together with rituximab (RTX) or in combination with RTX plus CD47-IgGσ (CD47). After 2 h, the phagocytic index was determined by fluorescence microscopy. Data points represent results obtained with individual preparations of macrophages from different donors (**P ≤ 0.01; ns, not significant; one-way ANOVA with Šidàk´s multiple comparisons test). **(C)** To verify antigen specific mode of action, LILRB1-IgGσ was compared with antibody HER2-IgGσ (IgGσ) in ADCP reactions. CFSE-labeled Carnaval cells were incubated in the absence (w/o) or in the presence of the indicated antibodies. Each antibody was applied at a concentration of 10 µg/ml. After 2 h, ADCP was analyzed by fluorescence microscopy. Data points represent phagocytic index values for individual preparations of macrophages from different donors (*n* = 4). Horizontal lines indicate mean values ± SD (*P ≤ 0.05; ns, not significant; one-way ANOVA with Šidàk´s multiple comparisons test). **(D)** Carnaval cells were labeled with CFSE and incubated with human M0 macrophages in the presence of the indicated antibodies (10 µg/ml). After 2 h, the supernatant was analyzed for residual lymphoma cells by live cell imaging and the percentage of residual lymphoma cells relative to the control reaction without added antibodies was calculated. Horizontal lines indicate mean values ± SD. (**P ≤ 0.01; *P ≤ 0.05; ns, not significant; one-way ANOVA and Šidàk´s multiple comparisons test; *n* = 5). **(E)** Granta 519 (*n* = 8), MEC2 (*n* = 10), DG-75 (*n* = 6), and SU-DHL-4 (*n* = 5) lymphoma cells were analyzed as target cells for RTX and anti-LILRB1 and CD47 antibodies. Target cells were labeled with CFSE and co-cultured with human M0 macrophages (E:T cell ratio: 1:2) in the absence (w/o) or in the presence of the indicated antibodies (10 µg/ml) for 2 h prior to analysis by fluorescence microscopy. In individual experiments, HER2-IgGσ (IgGσ) and trastuzumab (IgG1) were analyzed as controls. Data points represent phagocytic index values for macrophages from individual donors. Horizontal lines indicate mean values ± SD (*P ≤ 0.05; ns, not significant; one-way ANOVA with Šidàk´s multiple comparisons test). **(F)** The relative fold improvement in the extent of ADCP achieved by addition of LILRB1-IgGσ to RTX plus CD47-IgGσ over ADCP induced by the combination of RTX plus CD47-IgGσ only was calculated using phagocytic index values for different target cell lines as determined in **(B)**, **(C)** and **(E)**. Bars represent mean values ± SD. **(G)** Obinutuzumab (Obi) was analyzed in combination with LILRB1-IgGσ and CD47-IgGσ for induction of ADCP using a fluorescence microscopy based assay as described above. Granta 519 cells were used as target cells and human M0 macrophages were effector cells. Data points represent phagocytic index values for individual macrophage preparations from seven different donors. Horizontal lines represent mean values ± SD. (*P ≤ 0.05; **P ≤ 0.01; one-way ANOVA with Šidàk´s multiple comparisons test). **(H)** An IgA2 variant of rituximab (RTX-IgA2) was analyzed alone or in combination with CD47-IgGσ and/or LILRB1-IgGσ antibodies in ADCP assays with Carnaval cells. ADCP was analyzed as described above by fluorescence microscopy. As a control, an IgA2 version of cetuximab was employed (IgA2). *P ≤ 0.05; ns, not significant; one-way ANOVA with Šidàk´s multiple comparisons test (*n* = 7).

MCSF-generated M0 macrophages expressed significant amounts of the HLA class I receptors LILRB1 and LILRB2 (**Figure 1D**). In an attempt to further enhance ADCP by blocking the anticipated inhibitory function of HLA class I receptors, Fc-silent antibodies against LILRB1 and LILRB2 (LILRB1-IgGσ and LILRB2-IgGσ, respectively) were generated (**Supplementary Figures 1A, B**). Gel filtration revealed that the antibodies were monomeric and no significant multimers or higher molecular weight aggregates were detectable (**Supplementary Figure 1C**). The antigen specific binding of both antibodies was confirmed by flow cytometry using CHO-K1 cells transiently transfected with either LILRB1 or LILRB2 cDNA expression constructs, showing that the two antibodies exerted the expected binding profile (**Supplementary Figure 3**). Moreover, both LILRB1-IgGσ and LILRB2-IgGσ were able to block binding of soluble HLA class I molecules to LILRB1 and LILRB2 transfected CHO cells, respectively (**Figure 2A**). To analyze, whether the antibody blockade of LILRB1 and LILRB2 translated in enhanced ADCP, initial experiments with M0 macrophages and Carnaval target cells were performed. As a result, although not being effective alone or when combined with rituximab only, the LILRB1-IgGσ antibody significantly enhanced ADCP when applied together with both rituximab and CD47-IgGσ (**Figure 2B**). In contrast, no improvements in ADCP were observed by LILRB2 blockade, neither when LILRB2-IgGσ was applied alone nor in combination (**Figure 2B**). To demonstrate the antigen-specific mode of action of LILRB1-IgGσ, an Fc-silent control antibody, HER2-IgGσ, was combined with rituximab and CD47-IgGσ in ADCP reactions (**Figure 2C**). Whereas again LILRB1-IgGσ significantly improved ADCP, the HER2-IgGσ control antibody did not mediate any effects. Phagocytosis was also not observed when the LILRB1-IgGσ and CD47-IgGσ antibodies were applied in the absence of rituximab (**Figure 2C**), indicating that disruption of both signaling pathways was not sufficient in the absence of an activating signal. In addition, the direct comparison of the two triple combinations consisting of rituximab and CD47-IgGσ plus either LILRB1-IgGσ or LILRB2-IgGσ revealed significant differences between the two treatment groups, further demonstrating the benefits of the antigen-specific blockade of LILRB1 (**Supplementary Figure 4A**). In agreement with these findings, the application of the triple antibody combination consisting of rituximab, CD47-IgGσ plus LILRB1-IgGσ resulted in an enhanced depletion of Carnaval target cells, as analyzed by determining the residual remaining target cells using the IncuCyte^®^ live cell imaging system (**Figure 2D**).

LILRB1-IgGσ revealed the potential also to enhance ADCP of other lymphoma cell lines. In experiments with both Granta 519 and MEC2 cells LILRB1-IgGσ was effective when applied in a triple combination with rituximab and CD47-IgGσ (**Figure 2E**). Remarkably, rituximab plus the dual checkpoint blockade of LILRB1 and CD47 resulted in profound phagocytosis of DG-75 cells, which hardly responded to the treatment with rituximab plus CD47-IgGσ. Yet, ADCP of SU-DHL-4 cells was not significantly improved further, which might be explained by their high susceptibility to ADCP (**Figure 2E**). Thus, among the analyzed cell lines, the highest fold improvement in ADCP by inclusion of LILRB1-IgGσ was achieved with DG-75 cells (**Figure 2F**), which had the lowest CD20 expression levels (**Figure 1B**). In contrast, LILRB2 blockade was not effective in enhancing ADCP of MEC2 and DG-75 cells (**Supplementary Figure 4B**). Using DG-75 target cells, the potency of LILRB1 blockade relative to LILRB2 blockade was further demonstrated in a direct comparison of the two respective antibodies in combination with rituximab and CD47-IgGσ (**Supplementary Figure 4C**). Therefore, we focused on the LILRB1-IgGσ antibody in subsequent experiments. To analyze the effects of the dual checkpoint blockade with a different CD20 antibody, ADCP was investigated in combinations with the Fc glyco-engineered CD20 antibody obinutuzumab using Granta 519 target cells. As observed in experiments with rituximab, LILRB1-IgGσ demonstrated efficacy and enhanced ADCP when applied together with obinutuzumab and CD47-IgGσ (**Figure 2G**). Next, the efficacy of LILRB1 blockade was analyzed in combinations with an IgA2 isotype switch variant of rituximab (RTX-IgA2; **Figure 2H**). Using Carnaval target cells, RTX-IgA2 induced ADCP, and its efficacy was enhanced by combination with CD47-IgGσ. Importantly, LILRB1-IgGσ further potentiated ADCP when added to the RTX-IgA2 and CD47-IgGσ combination.

**Figure 3:**
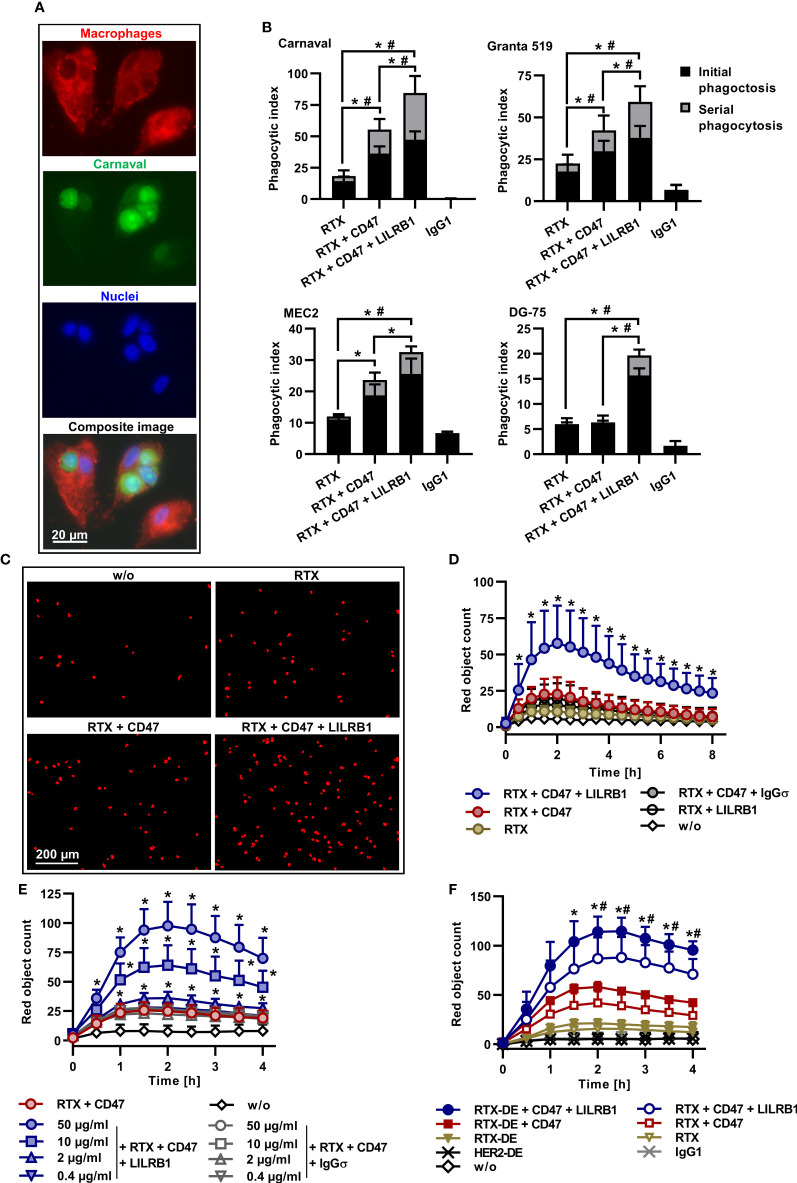
Serial ADCP, kinetics, dose-dependency and further enhanced ADCP by improved FcγR engagement. **(A)** CFSE-labeled Carnaval cells were incubated with Cell Brite™ Orange labeled human M0 macrophages in the presence of rituximab (RTX), CD47-IgGσ and LILRB1-IgGσ (E:T cell ratio: 1:2). Shown are three macrophages, having engulfed one or three Carnaval cells, or none. Nuclei were stained with NucBlue™. The bottom image represents a microscope composite image of the same cells viewed separately with the red, green and blue channels as indicated. **(B)** LILRB1-IgGσ (LILRB1) and CD47-IgGσ (CD47) antibodies promote serial ADCP of Carnaval (*n* = 9), Granta 519 (*n* = 8), MEC2 (*n* = 6) and DG-75 (*n* = 6) B-NHL cells. Phagocytic events by treatment with RTX, RTX + CD47-IgGσ, the triple combination of RTX + CD47-IgGσ + LILRB1-IgGσ or trastuzumab (IgG1) were assigned to uptake of the first lymphoma cell (initial phagocytosis) or to engulfment of subsequent cells (serial phagocytosis) and are presented as a proportion of phagocytic index values. Bars indicate mean values ± SEM. Statistically significant differences between treatment groups in initial (*) and serial (#) phagocytosis event values are indicated (P ≤ 0.05, two-way ANOVA with Tukey`s *post hoc* test). **(C)** ADCP induced by treatment with different antibodies (10 µg/ml) alone or in combination was analyzed by live cell imaging using pHrodo^®^ labeled DG-75 cells and human M0 macrophages (E:T cell ratio: 1:2). For analysis, cells were imaged at different time points and red fluorescent objects were counted. Shown are representative results for the control reaction without added antibodies (w/o), treatment with RTX, RTX + CD47-IgGσ (CD47) and RTX + CD47-IgGσ + LILRB1-IgGσ (LILRB1). The images were taken after 1.5 h (*n* = 9). **(D)** The kinetics of ADCP was analyzed by live cell imaging as described before. M0 macrophages from different donors were used as effector cells and DG-75 cells were applied as targets. Data points represent means ± SD of red object counts per image (*n* = 9; w/o, without added antibody). Statistically significant (P ≤ 0.05) differences between treatment groups RTX + CD47-IgGσ vs. RTX + CD47-IgGσ + LILRB1-IgGσ are indicated (*P ≤ 0.05; two-way ANOVA with Fisher´s LSD test). **(E)** PHrodo^®^ labeled DG-75 lymphoma cells were incubated with human M0 macrophages from different donors (*n* = 4) in the presence of RTX and CD47-IgGσ (each at a constant concentration of 10 µg/ml) plus LILRB1-IgGσ at varying concentrations. HER2-IgG2σ (IgGσ) was used as an isotype control. ADCP was analyzed over 4 h by live cell imaging. Data points represent means ± SD of red object count per image. Statistically significant differences between LILRB1-IgGσ and HER2-IgGσ treatments are indicated (*P ≤ 0.05, two-way ANOVA with Fisher´s LSD test). **(F)** RTX and an Fc-engineered RTX variant with enhanced FcγR binding (RTX-DE) were analyzed either alone or in combination with CD47-IgGσ and LILRB1-IgGσ as indicated using pHrodo^®^ labeled DG-75 cells and human M0 macrophages. Trastuzumab (IgG1) and its Fc-engineered version HER2-DE served as controls. ADCP was determined by live cell imaging analysis. Data points represent means of red object counts per image ± SD (*n* = 4). *, statistically significant differences between RTX-DE + CD47-IgGσ + LILRB1-IgGσ vs. RTX-DE + CD47-IgGσ; #, statistically significant differences between RTX-DE + CD47-IgGσ + LILRB1-IgGσ vs. RTX + CD47-IgGσ + LILRB1-IgGσ; P ≤ 0.05, two-way ANOVA with Fisher´s LSD test.

Individual macrophages are able to engulf multiple tumor target cells. Indeed, the analysis by fluorescence microscopy demonstrated that serial ADCP of Carnaval cells by M0 macrophages occurred in particular when rituximab was combined with CD47 and anti-LILRB1 antibodies (**Figure 3A**). To quantify the relative contribution, phagocytic events were assigned to uptake of the first lymphoma cell (initial phagocytosis) or to engulfment of subsequent cells (serial phagocytosis; **Figure 3B**). Serial phagocytosis was observed with Carnaval, Granta 519, MEC2 and, to a lesser extent, with DG-75 cells, and was promoted by co-treatment with CD47-IgGσ and LILRB1-IgGσ antibodies. Especially with more ADCP sensitive Carnaval and Granta 519 cells, increases in extent of ADCP by co-blockade of LILRB1 and CD47 were attributed to enhanced phagocytic activities of individual macrophages engulfing multiple target cells. The differences in the occurrence of serial phagocytosis of Carnaval and DG-75 cells was further evidenced by grouping macrophages according to the numbers of engulfed target cells (**Supplementary Figure 5**).

To analyze kinetics of ADCP induction, live cell imaging experiments were performed using DG-75 cells as targets (**Figures 3C, D**). Again, ADCP by rituximab was enhanced by dual checkpoint blockade of CD47 and LILRB1. ADCP occurred rapidly and reached a peak after 2 h. To analyze the dose dependent mode of action of LILRB1-IgGσ, rituximab and CD47-IgGσ were complemented with varying concentrations of either LILRB1-IgGσ or the control antibody HER2-IgGσ (**Figure 3E**). As a result, ADCP augmented with increasing concentrations of LILRB1-IgGσ while HER2-IgGσ was not effective.

Improving FcγR engagement by Fc engineering has been shown to enhance ADCP (21). To analyze, whether ADCP could be further potentiated, an Fc-engineered variant of rituximab (RTX-DE) was generated by introducing the amino acid substitutions S239D/I332E (**Supplementary Figure 1**). This modification enhances the antibody`s affinity to activating FcγRI, FcγRIIA and FcγIIIA and improves ADCP and ADCC (51). RTX-DE was compared with rituximab in the absence or presence of immune checkpoint inhibitors in ADCP assays using DG-75 cells by live cell imaging (**Figure 3F**). As a result, rituximab and RTX-DE were only marginally effective, in agreement with previous findings for this cell line. By combination of both antibodies with CD47-IgGσ ADCP was enhanced. Importantly, the triple antibody combination consisting of RTX-DE, LILRB1-IgGσ and CD47-IgGσ was more effective than the triple antibody combination containing rituximab. This demonstrates the impact of efficient FcγR engagement and indicates that ADCP can be promoted further by improving the affinity of the tumor targeting antibody to activating FcγR.

**Figure 4:**
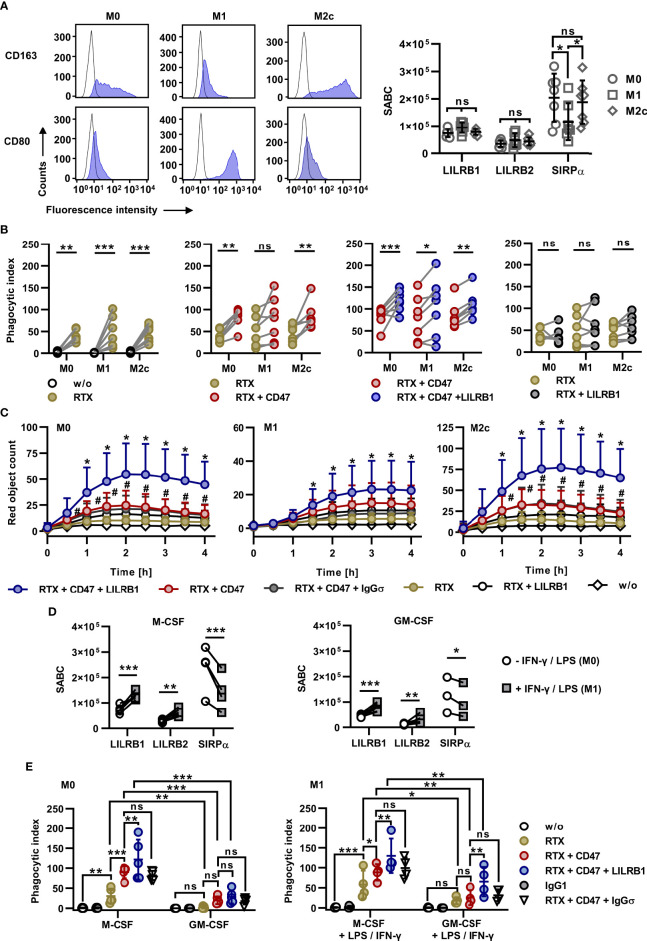
Efficacy of dual checkpoint blockade of CD47 and LILRB1 with differentially polarized macrophages. **(A)** *Left panel:* Monocytes were differentiated with M-CSF (M0 macrophages), GM-CSF, LPS and IFN-γ (M1 macrophages) or M-CSF and IL-10 (M2c macrophages) and stained with PE-conjugated antibodies against CD163, CD80 (blue shaded peaks) or an isotype control antibody (black outlined peaks). Cell surface expression was analyzed by flow cytometry. One representative experiment is shown (*n* = 3). *Right panel:* Cell surface expression of LILRB1, LILRB2 and SIRPα by M0, M1 and M2c macrophages was analyzed by calibrated flow cytometry. Data points indicate specific antibody binding capacity (SABC) for macrophage preparations from individual donors. Horizontal lines represent mean values ± SD (*n* = 7). *P ≤ 0.05; ns, not significant; two way ANOVA and Tukey`s *post hoc* test. **(B)** Macrophages were differentiated in parallel from monocytes from seven donors towards M0, M1 or M2c phenotypes and analyzed in fluorescence microscopy-based ADCP assays using CFSE-labeled Carnaval cells (E:T cell ratio: 1:2). Efficacy was determined for rituximab (RTX) vs. phagocytosis in the absence of an antibody (w/o, *first graph*), RTX + CD47-IgGσ (CD47) vs. RTX (*second graph*), and RTX + CD47-IgGσ + LILRB1-IgGσ (LILRB1) vs. RTX + CD47-IgGσ (*third graph*) and RTX + LILRB1-IgGσ vs. RTX (*fourth graph*). Antibodies were applied at a concentration of 10 µg/ml. Statistically significant differences are indicated (*P ≤ 0.05; **P ≤ 0.01; ***P ≤ 0.001; ns, not significant; two-way ANOVA with Šidàk´s multiple comparisons test). **(C)** Live-cell imaging analysis of ADCP by M0, M1 and M2c macrophages in the presence of the indicated antibodies (each at a concentration of 10 µg/ml). HER2-IgGσ (IgGσ) was used in control reactions. Target cells were pHrodo^®^ labeled DG-75 cells (E:T cell ratio: 1:2). Data points represent means ± SD of the red object count per image of independent experiments using macrophages from six different donors that were polarized in parallel towards M0, M1 or M2c phenotypes (w/o, without antibody; #, statistically significant differences between RTX + CD47-IgGσ vs. RTX; *, statistically significant differences between RTX + CD47-IgGσ + LILRB1-IgGσ vs. RTX + CD47-IgGσ, two-way ANOVA and Fisher´s LSD test; P ≤ 0.05). **(D)** Macrophages were differentiated from peripheral monocytes in the presence of either M-CSF (*left graph*) or GM-CSF (*right graph*) for six days. Cells were left untreated or stimulated with IFN-γ and LPS for additional 48 h and analyzed for cell surface expression of LILRB1, LILRB2 and SIRPα by calibrated flow cytometry. Data points represent the specific antibody binding capacities (SABC) that were determined for individual macrophage preparations. Statistically significant differences between groups treated with IFN-γ and LPS and the control groups are indicated (*P ≤ 0.05; **P ≤ 0.01; ***P ≤ 0.001; two-way ANOVA with Fisher´s LSD test). **(E)** Macrophages were differentiated with M-CSF or GM-CSF and analyzed without further stimulation (M0 macrophages; *left graph*; *n* = 5) or after polarization with LPS and IFN-γ (M1 macrophages; *right graph*; *n* = 4) in 2 h ADCP assays using CFSE-labeled Carnaval cells and the different antibodies as indicated. ADCP was analyzed by fluorescence microscopy. Data points indicate phagocytic index values for macrophages from individual donors. Horizontal lines represent mean values ± SD (*P ≤ 0.05; **P ≤ 0.01; ***P ≤ 0.001; ns, not significant; two-way ANOVA and Fisher´s LSD test).

To analyze the impact of HLA class I receptors on ADCP by polarized macrophages, macrophages were differentiated towards M1 and M2c phenotypes using GM-CSF, LPS and IFN-γ or M-CSF and IL-10, respectively. Macrophage polarization was verified by determining expression levels of the M1 and M2 marker antigens CD80 and CD163, respectively (**Figure 4A**) The analysis of LILRB1 and LILRB2 cell surface expression revealed that M1 and M2c macrophages expressed both receptors at similar levels as M0 macrophages (**Figure 4A**). However, the expression of SIRPα was reduced in M1 macrophages. M1 and M2c macrophages were then analyzed as effector cells for combinations of rituximab, CD47-IgGσ and LILRB1-IgGσ in ADCP assays with Carnaval cells and compared to non-polarized, M-CSF differentiated M0 macrophages from the same donors (**Figure 4B**). As a result, rituximab was effective in inducing ADCP with different macrophage populations (**Figure 4B**). In experiments with M0 and M2c macrophages, CD47-IgGσ augmented rituximab-mediated ADCP significantly. With M1 macrophages a similar trend was observed, but statistical significance was not reached. Importantly, LILRB1-IgGσ enhanced ADCP not only by M0 macrophages, but also by polarized M1 or M2c macrophages when applied in addition to rituximab and CD47-IgGσ. As observed with M0 macrophages, LILRB1 blockade alone was not sufficient to enhance ADCP by rituximab with both M1 and M2c macrophages (**Figure 4B**). Moreover, ADCP by differentially polarized macrophages was analyzed by live cell imaging using DG-75 as target cells (**Figure 4C**). These experiments demonstrated improved ADCP of DG-75 cells by rituximab when combined with both LILRB1-IgGσ and CD47-IgGσ antibodies irrespective of the macrophage polarization status. In agreement with results obtained with M0 macrophages, LILRB2-IgGσ was not effective when M1 or M2c macrophages were analyzed (**Supplementary Figures 4D, E**). Furthermore, M0 macrophages were differentiated from monocytes in the presence of either M-CSF or GM-CSF and then polarized to M1 macrophages using LPS and IFN-γ. Interestingly, cell surface expression of both LILRB1 and LILRB2 was upregulated upon polarization with LPS and IFN-γ, while SIRPα expression was reduced (**Figure 4D**). In ADCP experiments using fluorescence microscopy, non-activated M0 macrophages differentiated with GM-CSF were not effective in comparison with macrophages differentiated with M-CSF (**Figure 4E**). Pre-treatment with LPS/IFN-γ improved ADCP by GM-CSF macrophages, in particular when the triple combination consisting of RTX, CD47-IgGσ and LILRB1-IgGσ was applied. LPS/IFN-γ stimulation also slightly enhanced the ADCP activity of M-CSF macrophages, which were superior to GM-CSF macrophages for each antibody treatment also upon M1 polarization with LPS/IFN-γ. Of note, the triple combination consisting of RTX, LILRB1-IgGσ and CD47-IgGσ was most efficacious in experiments with both macrophage populations.

**Figure 5:**
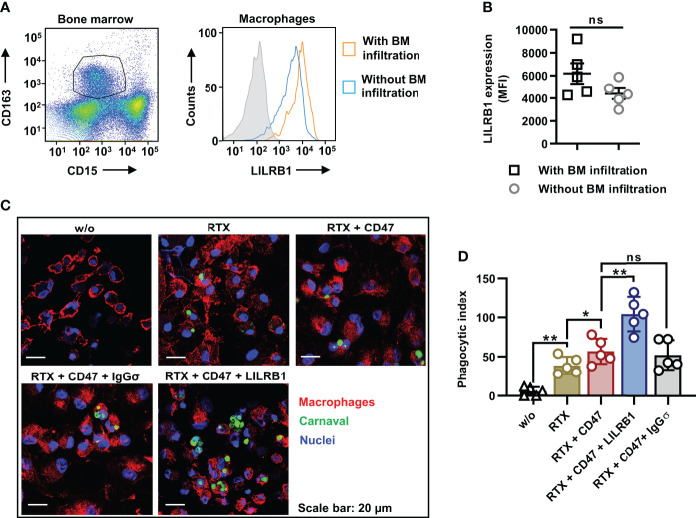
Dual checkpoint blockade of CD47 and LILRB1 enhances ADCP by LAM from DLBCL patients. **(A)** Lymphoma associated macrophages (LAM) in the MNC fraction from bone marrow (BM) samples of DLBCL patients with BM infiltration (*n* = 5) were defined as CD163-positive/CD15-negative cells by staining with Brilliant Violet 421-conjugated CD163 and Brilliant Violet 510-labeled CD15 antibodies and flow cytometry analysis (*left histogram*). Macrophages were gated, and analyzed for the cell surface expression of LILRB1, which was detected using an APC-antibody-conjugate (*right histogram*). For comparison, BM macrophages from DLBCL patients without BM infiltration were analyzed in parallel. The gray histogram indicates the isotype control. **(B)** The graph summarizes results for the LILRB1 expression according to median fluorescent intensities for BM macrophages from individual patients with (LAM) or without BM infiltration. Horizontal lines show mean values, error bars indicate SD (ns, not significant; P = 0.129). **(C)** CD163-positive/CD15-negative LAM were purified by fluorescence activated cell sorting. In ADCP reactions, LAM were plated and incubated with Carnaval cells labeled with Cytolight Rapid Green (E:T cell ratio: 1:1) in the absence (w/o) or in the presence of the antibodies rituximab (RTX), LILRB1-IgGσ (LILRB1), CD47-IgGσ (CD47) or HER-IgGσ (IgGσ; each at a concentration of 1 µg/ml), as indicated. After 2 h, cells were stained with an APC-conjugated CD11b antibody, fixed and stained with DAPI. ADCP was analyzed using a confocal microscope at x630 magnification. Images from one representative experiment are shown (*n* = 5). **(D)** The graph summarizes the phagocytic index values for ADCP of Carnaval cells by LAM upon treatment with different antibodies as indicated. Data points represent the phagocytic index values for LAM isolated from individual patients (*n* = 5). Bars indicate mean values ± SD (*P ≤ 0.05; **P ≤ 0.01; ns, not significant; one way ANOVA with Šidàk´s multiple comparisons test).

In an effort to analyze the potential of LILRB1 and CD47 co-blockade to enhance the ADCP activity also of lymphoma-associated macrophages (LAM), MNC were prepared from BM samples from DLBCL patients with BM infiltration (**Figure 5**). Of note, flow cytometry analysis revealed a strong expression of LILRB1 by LAM, which were defined as CD163-positive/CD15-negative cells (**Figures 5A, B**). For comparison, also LILRB1 expression by BM macrophages from DLBCL patients without BM infiltration was assessed (**Figures 5A, B**). A trend towards a higher LILRB1 expression in the mean was found in LAM, although the observed differences were not statistically significant. For subsequent ADCP analysis, CD163-positive/CD15-negative LAM were purified by fluorescence activated cell sorting and analyzed without further manipulation as effector cells for rituximab and the antibody combinations using Carnaval target cells (**Figures 5C, D**). Importantly, a considerable further improvement in ADCP was observed when the antibody triple combination consisting of rituximab, CD47-IGgσ and LILRB1-IgGσ was applied relative to the combination treatment with RTX and CD47-IgGσ only.

**Figure 6:**
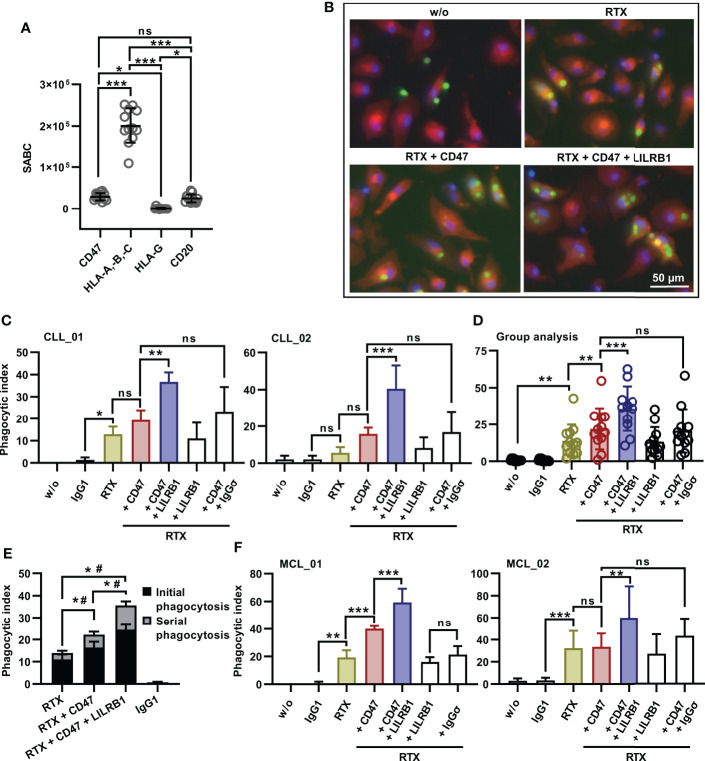
Enhanced ADCP of patient-derived CLL or MCL cells by dual checkpoint blockade of CD47 and LILRB1. **(A)** Cell surface expression levels of CD20, CD47, HLA-A,-B,-C and HLA-G by patient CLL cells was analyzed by calibrated flow cytometry. Data points represent specific antibody binding capacity (SABC) for individual patient samples. The horizontal lines indicate mean values ± SD (*P ≤ 0.05; ***P ≤ 0.001; ns, not significant; one-way ANOVA with Tukey’s multiple comparison test. **(B)** CFSE-labeled CLL cells (patient CLL_05) were incubated with M0 macrophages (labeled with Cell Brite™ Orange cytoplasmic membrane dye, E:T cell ratio: 1:2) in the absence (w/o) or in the presence of the antibodies rituximab (RTX), CD47-IgGσ (CD47) and LILRB1-IgGσ (LILRB1; each at a concentration of 10 µg/ml) as indicated for 2 h. Nuclei were stained with NucBlue™. ADCP was analyzed by fluorescence microscopy. **(C)** CLL cells from patients CLL_01 and CLL_02 were labeled with CFSE and incubated with M0 macrophages without (w/o) or with antibodies trastuzumab (IgG1), rituximab (RTX), LILRB1-IgGσ (LILRB1), CD47-IgGσ (CD47) or HER2-IgGσ (IgGσ) as indicated (each at a concentration of 10 µg/ml) for 2 h. ADCP was analyzed by fluorescence microscopy. Bars represent mean phagocytic index values ± SD from three independent experiments using macrophages from different donors (*P ≤0.05; **P ≤ 0.01; ***P ≤ 0.001; ns, not significant; one-way ANOVA with Fisher`s LSD test). **(D)** Group analysis of ADCP summarizing results obtained with CLL cells from 12 different patients as target cells. ADCP was determined for the indicated antibodies or their combinations as described in **(C)**. Data points represent mean phagocytic index values for individual patient samples as determined in independent experiments using macrophages from different donors, as illustrated in Suppl. Figure 6. Bars indicate overall mean phagocytic index values ± SD (**P ≤ 0.01; ***P ≤ 0.001; ns, not significant; one-way ANOVA with Šidàk´s multiple comparisons test). **(E)** LILRB1 and CD47 immune checkpoint blockade promotes serial ADCP of patient CLL cells by human M0 macrophages. Phagocytic events were assigned to phagocytosis of the first CLL cell (initial phagocytosis) or to engulfment of subsequent CLL cells (serial phagocytosis) and are presented as a proportion of phagocytic index values. Bars represent mean values ± SEM. Statistically significant differences between treatment groups in initial phagocytosis (*) and serial phagocytosis (#) are indicated (P ≤ 0.05; two-way ANOVA and Tukey`s multiple comparison test; *n* = 12). **(F)** MCL cells were isolated from two patients, labeled with CFSE and analyzed as target cells for M0 macrophages in the presence of the indicated antibodies (each at a concentration of 10 µg/ml) by fluorescence microscopy as described in **(C)**. Data points represent mean phagocytic index values ± SD from three (MCL_01) or six (MCL_02) independent experiments using macrophages from different donors (**P ≤0.01; ***P ≤ 0.001; ns, not significant; one-way ANOVA with Fisher`s LSD test).

Because cell lines do not reflect the clinical heterogeneity of patients, the dual checkpoint blockade of CD47 and LILRB1 was investigated using tumor cells from patients. CLL cells were enriched from the peripheral blood of twelve patients. The analysis of cell surface antigen expression levels revealed pronounced expression of CD20, CD47 and classical HLA class I molecules in all CLL patient samples, in contrast to HLA-G, which was hardly detected (**Figure 6A**). Patient cells were then analyzed in ADCP experiments using M0 macrophages from healthy donors and fluorescence microscopy (**Figure 6B**). CD47-IgGσ enhanced ADCP by rituximab in the majority of individual patient samples, with exception of samples CLL_04 and CLL_10, in which CD47 blockade did not improve ADCP despite considerable CD20 and CD47 expression (**Supplementary Figure 6**). Of note, dual checkpoint blockade of CD47 and LILRB1 enhanced ADCP by rituximab further and LILRB1-IgGσ amplified the degree of ADCP when combined with rituximab and CD47-IgGσ. Thus, in all cases analyzed the triple antibody combination of rituximab, LILRB1-IgGσ and CD47-IgGσ was more efficacious than the double antibody combination consisting of rituximab and CD47-IgGσ (**Figure 6C**; **Supplementary Figure 6**). Sample group analysis revealed statistical significance of the observed differences between different treatment groups (**Figure 6D**). The application of LILRB1-IgGσ with rituximab in the absence of the CD47 antibody did not translate into higher ADCP (**Figure 6D**). With all CLL cell samples tested, LILRB2 blockade was not effective (unpublished data). Further analysis revealed that, as also observed with cell line experiments, serial ADCP events occurred and were observed more frequently upon treatment with the antibody triple combination (**Figure 6E**). Finally, dual checkpoint blockade of CD47 and LILRB1 was tested with MCL tumor cells isolated from two patients (**Figure 6F**). Similar to CLL cells, rituximab-mediated ADCP was augmented by co-blockade of CD47 and LILRB1 with LILRB1-IgGσ providing an amplifying effect.

## Discussion

Analysis of different B-NHL cell lines revealed that the expression ratio of CD20 to HLA class I molecules determined the sensitivity to ADCP by the combination of rituximab and a CD47 blocking antibody. We thus investigated the impact of blocking the HLA class I receptors LILRB1 and LILRB2 on CD20 antibody-mediated ADCP with or without concomitant masking of CD47 using Fc-silent antibodies. While the anti-LILRB2 antibody was not effective, the anti-LILRB1 antibody enhanced ADCP considerably, but strictly required simultaneous CD47 blockade and the presence of a tumor targeting CD20 antibody to become effective. Thus, the dual checkpoint blockade of CD47 and LILRB1 enhanced ADCP by rituximab, obinutuzumab, an Fc-engineered variant of rituximab and an IgA2 version of rituximab. The LILRB1 co-blockade promoted serial uptake of lymphoma cells, and demonstrated efficacy in both B-NHL cell lines and freshly isolated MCL or CLL cells from patients.

In recent years, impressive clinical results were obtained with the application of adaptive immune checkpoint inhibitors to establish T cell tumor immunity (6, 52). Regarding B cell lymphomas, promising results were observed with immune checkpoint inhibitors targeting the PD-1/PD-L1 axis in the treatment of classical Hodgkin lymphoma with response rates exceeding 70% (53). Yet, response rates with immune checkpoint monotherapies in the majority of B-NHL types including DLBCL, follicular lymphoma and CLL were unsatisfactory (53, 54). For example, a phase II trial with the anti-PD-1 antibody nivolumab in patients with relapsed or refractory DLBCL revealed response rates of only 10%, and no objective responses were observed in relapsed CLL patients upon treatment with the anti-PD-1 antibody pembrolizumab (55, 56). The application of immune checkpoint inhibitors in combination with other therapies such as R-CHOP chemo-immunotherapy may hold promise (53, 57), but will require further investigation. Limitations arise when tumors create an immune hostile microenvironment and exert insufficient immunogenicity. In this situation, the recruitment of innate immune cells, which in the tumor microenvironment contribute to tumor immunity, may be an alternative (7, 58). Particularly the immune checkpoint blockade in myeloid cells has gained increasing attention and encouraging clinical results were obtained by the combination treatment with rituximab and the CD47 antibody magrolimab in B-NHL patients (28).

As demonstrated here, the blockade of LILRB1 in addition to CD47 may offer a possibility further enhancing rituximab-mediated ADCP of lymphoma cells. However, even when both antigens were blocked, a considerable variation between different target cells in susceptibility to ADCP was observed. This may reflect the complex regulation of phagocytosis, which is governed by an interplay between activating and inhibitory receptors. Thus, ‘Don´t Eat Me!’ functions have been demonstrated for several antigens including programmed death ligand 1, CD24, adipocyte plasma membrane-associated protein, and signaling lymphocyte activation molecule (SLAM) family members (10, 59–61). Cognate receptors may cooperate with LILRB1 and SIRPα, and the expression of ligands for such receptors may contribute to less ADCP sensitive phenotypes of for example DG-75 cells or CLL cells from certain patients in our study. In addition, the engagement of pro-phagocytic receptors such as prolow-density lipoprotein receptor-related protein 1, CD137, CD11b and the currently discussed SLAMF7, as well as additional target cell characteristics such cell size, shape or rigidity may be important (10, 62–65).

The observation that the anti-LILRB1 antibody required simultaneous blockade of the CD47-SIRPα axis suggests that SIRPα exerts a dominant inhibitory role in the regulation of ADCP. Whether differences between receptors in signaling pathways exist, leading to impairment of phagocytosis at different stages of phagocytosis initiation, still needs to be investigated. Both receptors signal *via* ITIM in their intracellular domains. Initiation of SIRPα signaling suppresses phagocytosis by reducing contacts between macrophages and target cells through inhibition of integrin activation, inhibition of cytoskeleton rearrangement by dephosphorylation of myosin IIA, and inactivation of neighboring FcγR through dephosphorylation of ITAM (66–68). The molecular pathways by which LILRB1 regulates phagocytosis by macrophages have not been clarified, yet impaired tyrosine phosphorylation of FcγR chain and inhibition of intracellular calcium mobilization was demonstrated upon co-ligation of FcγRI and LILRB1 (38). Of note, the dual checkpoint blockade of CD47 and LILRB1 alone was not sufficient to trigger phagocytosis and the presence of an FcR engaging CD20 antibody was required. Thus, in this approach, the specificity for phagocytic target cell elimination is maintained and is pre-defined by the tumor targeting antibody bearing a functional Fc domain to provide an activating signal.

In contrast to LILRB1, no benefits were obtained by blockade of LILRB2 - though the receptor was expressed by monocyte-derived macrophages at similar levels as LILRB1, the antibody used in this study blocked receptor binding of HLA molecules in agreement with previous findings (44), and interference with FcγR signaling by LILRB2 signaling has been demonstrated previously (38). However, structural differences in binding exist in that LILRB1 binds only β2-microglobulin associated HLA molecules, while LILRB2 also binds free forms (35, 69). In addition, the receptors differ in their affinity to individual HLA alleles (69). We assume that the quality of receptor engagement, the affinity to HLA molecules or the initiation and strength of the individual receptor’s intracellular signaling pathway may contribute to the observed differences. In addition, it cannot be fully excluded that the particular antibody clone employed in these experiments exerted agonistic functions and activated LILRB2 signaling in parallel to inhibition of ligand binding.

Enhanced ADCP by co-blockade of LILRB1 and CD47 was demonstrated for combinations with CD20-specific IgG antibodies. Improved ADCP was observed not only in combinations with the native IgG1 molecule rituximab, but also with the Fc glyco-engineered antibody obinutuzumab and the Fc-protein engineered rituximab variant RTX-DE. Moreover, co-inhibition of LILRB1 and CD47 was effective, when the antibodies were combined with an IgA2 variant of rituximab. IgA antibodies may hold potential for cancer immunotherapy. They are able to trigger myeloid effector cells including neutrophils, monocytes and macrophages by engagement of the IgA Fc receptor FcαRI. Previously it has been demonstrated that CD47 blockade enhances macrophage-mediated ADCP by CD20 IgA antibodies (49). As demonstrated in the present study, the efficacy can be further improved by co-blockade of LILRB1, suggesting that LILRB1 also regulates signaling by FcαRI.

Besides promoting ADCP, anti-LILRB1 antibodies may exert additional effector functions. Both antibody blockade of LILRB2 and genetic deletion of LILRB1 were shown to drive macrophage polarization towards an inflammatory M1 phenotype (36, 39). Therefore, the blockade of these receptors may facilitate to relieve immune suppression in the tumor microenvironment by shaping TAM or myeloid derived suppressor cells (58). In addition, enhanced engulfment of tumor cells may increase antigen presentation and promote T cell responses (10). In a murine tumor model, adaptive T cell responses were observed after treatment with CD47 antibodies (70). However, to unravel a role for LILRB1 in this context will require further investigation. In addition, LILRB1 and LILRB2 are expressed by other immune cell populations. For example, LILRB1 is expressed by T cells and a subpopulation of NK cells (32). Therefore, manipulation of LILRB1 may also promote cytotoxic functions by lymphocytes (71, 72). Regarding that NK cells may express SIRPα in certain situations even co-blockade of LILRB1 and CD47 may be effective in enhancing NK cell-mediated ADCC (73). In neutrophils, conflicting results on the expression of LILRB1 were published, but LILRB2 is displayed (32, 74). Albeit LILRB2-IgGσ lacked efficacy in enhancing ADCP by macrophages, it may be worth testing this antibody with neutrophils, in which blockade of the CD47-SIRPα axis was shown to enhance ADCC and trogoptosis (75).

To further advance the proposed concept animal studies will be required. However, this is a challenging issue, because LILRB1 is not expressed in mice and the murine receptor orthologue paired immunoglobulin-like receptor B does not react with HLA/β2M complexes (36). The generation of LILRB1 transgenic mice could offer an opportunity to study this. In a previous study, LILRB1 knock-in mice were established in the background of immune competent mice (76), but here immune deficient mice will be required to facilitate the engraftment of human lymphoma cells. Humanized mice in which human immune cells are established by transplantation of human CD34-positive progenitor cells could offer another option (77). However, the presence of human immune cells may hamper the co-engraftment of tumor cells and partial HLA matching may be required. In addition, co-existing murine macrophages, which respond to CD47 but not to anti-LILRB1 antibody blockade (36), may distort results, and an accompanying depletion of murine macrophages may be necessary. Yet, our *in vitro* results provide a rationale to undertake these efforts to realize such xenograft *in vivo* studies.

In conclusion, our preclinical *in vitro* results suggest potential of combining CD20, CD47 and anti- LIRB1 antibodies. LILRB1 blockade complemented CD47 inhibition and thus a dual checkpoint blockade of CD47-SIRPα and LILRB1-HLA class I interactions may have the potential to improve antibody therapy of lymphomas further by enhancing ADCP by macrophages. Therefore, combinations of tumor targeting antibodies with LILRB1 and either CD47 or SIRPα immune checkpoint inhibitors deserve further evaluation in animal models towards clinical application.

## Data availability statement

The original contributions presented in the study are included in the article/**Supplementary Material**. Further inquiries can be directed to the corresponding author.

## Ethics statement

The studies involving human participants were reviewed and approved by Ethics Committee of the faculty of medicine, LMU Munich. The patients/participants provided their written informed consent to participate in this study.

## Author contributions

Conceptualization, CK. Methodology, TZ, SL, MP, HB, AM and CK. Validation, TZ, SL and CK. Formal analysis, TZ, SL, IM, AM and CK. Investigation, TZ, SL, IM, RW, PH, TR, NB, CF, HB and CK. Resources, NT, JB, TH, OW, CW, MB-B, MP, HB, DS and AH. Writing - original draft preparation, TZ and CK. Writing - review and editing, SL, RW, TH, OW, MB-B, CW, TV, DS, HB and AH. Visualization, TZ, SL, HB and CK. Supervision, CK. All authors have read and agreed to the published version of the manuscript.

## Funding

This work was funded by research grants by the Deutsche Krebshilfe (70113524 and 70113533, to DS and CK), the Verein zur Förderung von Wissenschaft und Forschung an der Medizinischen Fakultät der Ludwig-Maximilians-Universität München (to CK) and the Deutsche José Carreras Leukämie-Stiftung to TH (DJCLS 10 R/2021).

## Acknowledgments

Irene Pauls, Lena Halang, and Fiona E. Rosmus are kindly acknowledged for expert technical assistance. The Deutsche José Carreras Leukämie-Stifung and the Deutsche Gesellschaft für Hämatologie und Medizinische Onkologie are gratefully acknowledged for the José Carreras-DGHO scholarship for doctoral research to TZ (05 PSD/2021). We also kindly thank the study participants.

## Conflict of interest

The authors declare that the research was conducted in the absence of any commercial or financial relationships that could be construed as a potential conflict of interest.

## Publisher’s note

All claims expressed in this article are solely those of the authors and do not necessarily represent those of their affiliated organizations, or those of the publisher, the editors and the reviewers. Any product that may be evaluated in this article, or claim that may be made by its manufacturer, is not guaranteed or endorsed by the publisher.
