# Supplementary material for: Dual checkpoint blockade of CD47 and LILRB1 enhances CD20 antibody-dependent phagocytosis of lymphoma cells by macrophages
Source: Front Immunol. 2022 Oct 27;13:929339. doi: 10.3389/fimmu.2022.929339 (PMC9647079; doi:10.3389/fimmu.2022.929339)
Supplement: Supplementary file 1 [file DataSheet_1.pdf]

Supplement

Supplementary Table 1: Statistically significant differences in the phagocytic index value and in the CD20 / HLA-A, -B, -C ratio between lymphoma cell lines[1]

Cell lines	Phagocytic index		CD20 / HLA-A, -B, -C ratio	
	p-value	Summary	p-value	Summary
Granta 519 vs. DG-75	0.0015	**	0.1214	ns
Granta 519 vs. MEC2	0.0221	*	0.9102	ns
Granta 519 vs. Carnaval	0.0043	**	0.0001	****
Granta 519 vs. SU-DHL-4	<0.0001	****	0.0001	****
DG-75 vs. MEC2	0.1703	ns	0.1463	ns
DG-75 vs. Carnaval	<0.0001	****	0.0001	****
DG-75 vs. SU-DHL-4	<0.0001	****	0.0001	****
MEC2 vs. Carnaval	<0.0001	****	0.0001	****
MEC2 vs. SU-DHL-4	<0.0001	****	0.0001	****
Carnaval vs. SU-DHL-4	<0.0001	****	0.0001	****

[1]Statistical analysis was performed with results depicted in Figure 1C using one-way ANOVA and Fisher`s LSD test (ns, not significant).

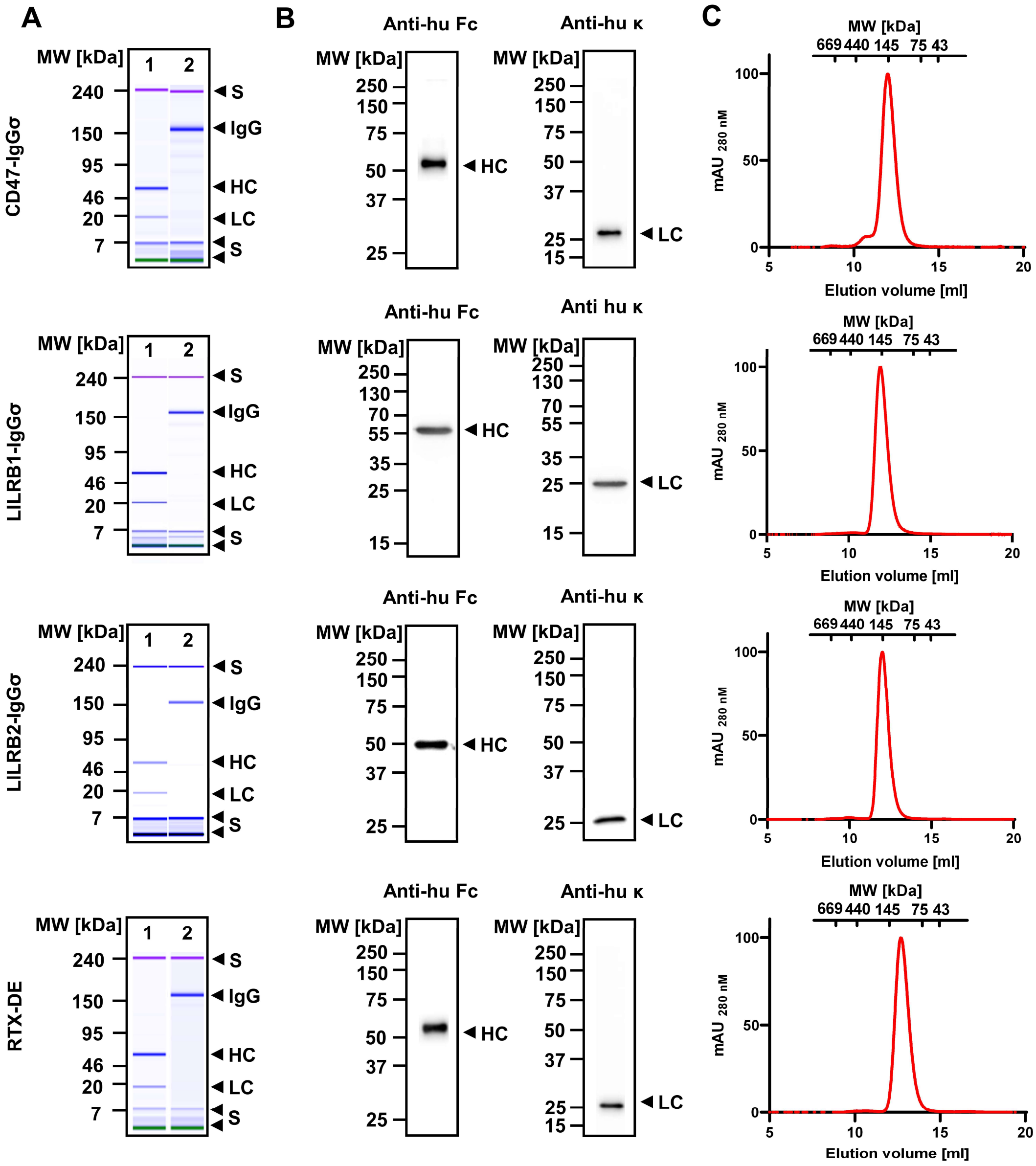

Supplementary Figure 1: Purity, integrity and homogeneity of recombinant antibodies. (A) The purified antibodies CD47-IgGσ, LILRB1-IgGσ, LILRB2-IgGσ and RTX-DE were analyzed by microfluidic chip electrophoresis under reducing (lane 1) or non-reducing (lane 2) conditions. Four microliters of antibody preparations were loaded. Distinct protein bands were assigned to separated antibody heavy chains (HC), light chains (LC) or disulfide-bridged IgG molecules according to apparent molecular weights (MW). Upper and lower system peaks are indicated (S). **(B)** For Western transfer experiments, 1 µg of purified antibodies was loaded on 12% (LILRB1-IgGσ) or 10% (CD47-IgGσ, LILRB2-IgGσ and RTX-DE) polyacrylamide gels, separated by sodium dodecyl sulfate polyacrylamide gel electrophoresis under reducing conditions, and transferred to nitrocellulose membranes. Antibody heavy (HC) and light chains (LC) were detected using horseradish-peroxidase-conjugated Fc-specific goat anti-human-IgG (anti-hu Fc) and goat anti-human κ light chain (anti-hu κ) antibodies, respectively. **(C)** Antibodies CD47-IgGσ, LILRB1-IgGσ, LILRB2-IgGσ and RTX-DE were analyzed by gel filtration by injecting 100 - 400 µg of proteins. Thyroglobulin (669 kDa), ferritin (440 kDa), conalbumin (75 kDa), ovalbumin (43 kDa, all purchased from Cytiva) and trastuzumab (145 kDa) were employed as molecular weight controls.

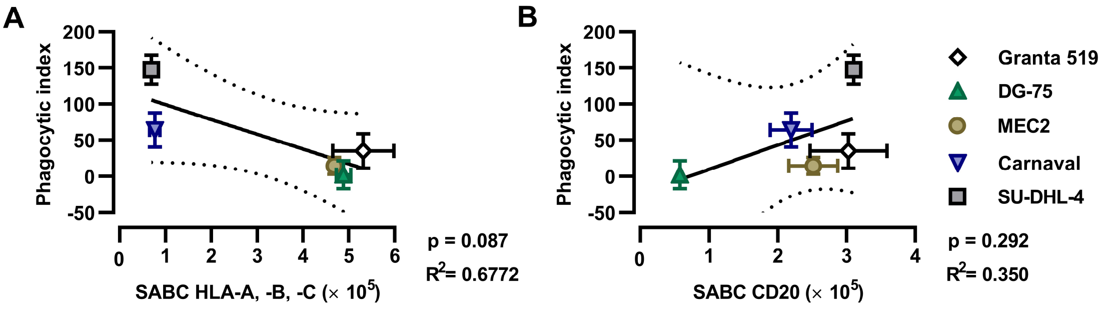

Supplementary Figure 2: Correlation analysis of HLA class I and CD20 expression levels and sensitivity to ADCP. The cell surface expression levels of CD20 and HLA-A, -B, -C molecules were determined for Granta 519, DG-75, MEC2, Carnaval and SU-DHL-4 cells by calibrated flow cytometry as illustrated in Figure 1. For each cell line, the specific antibody binding capacities (SABC) for HLA-A, -B, -C **(A)** and CD20 **(B)** were plotted against the calculated mean phagocytic index values for the treatment with RTX plus CD47-IgGσ. The solid line represents the best-fit curve, dotted lines indicate the 95% confidence interval.

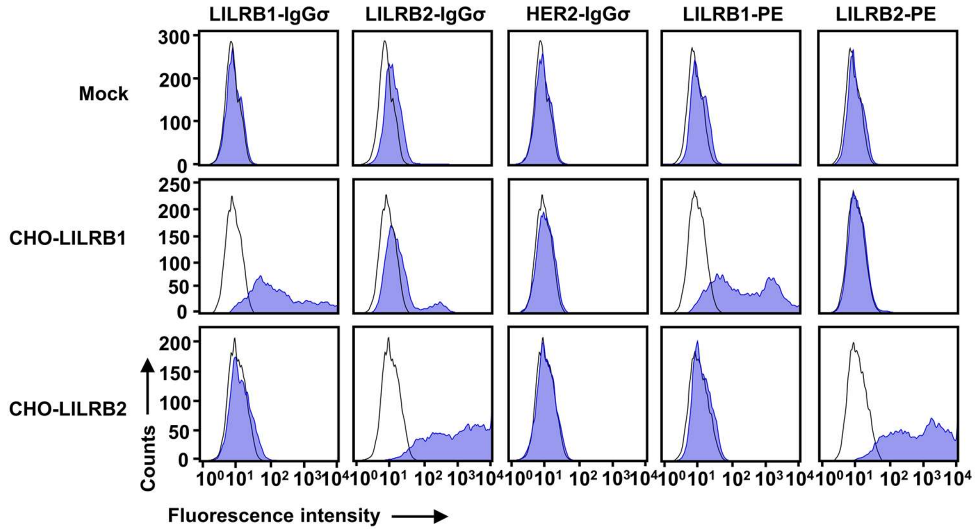

Supplementary Figure 3: Antigen-specific binding of LILRB1-IgGσ and LILRB2-IgGσ antibodies. CHO-K1 cells were transiently transfected with either LILRB1 or LILRB2 cDNA expression vectors. Thus generated CHO-LILRB1 and CHO-LILRB2 cells, respectively, as well as mock transfected CHO-K1 cells (Mock) were incubated in buffer only or in the presence of antibodies LILRB1-IgGσ, LILRB2-IgGσ or HER2-IgGσ, as indicated. Each antibody was analyzed at a concentration of 50 µg/ml. Secondary PE-conjugated $F(ab')_2$ fragments of goat anti-human Fcγ region antibodies were employed for detection. Purchased PE-conjugated anti-LILRB1 (LILRB1-PE) and anti-LILRB2 (LILRB2-PE) antibodies were used to verify efficient cell surface expression of LILRB1 and LILRB2 antigens, respectively. Antibody binding was analyzed by flow cytometry (black outlined peak: signal from buffer control, blue shaded peak: signals from antibodies, as indicated). Representative results from one out of three performed experiments are shown.

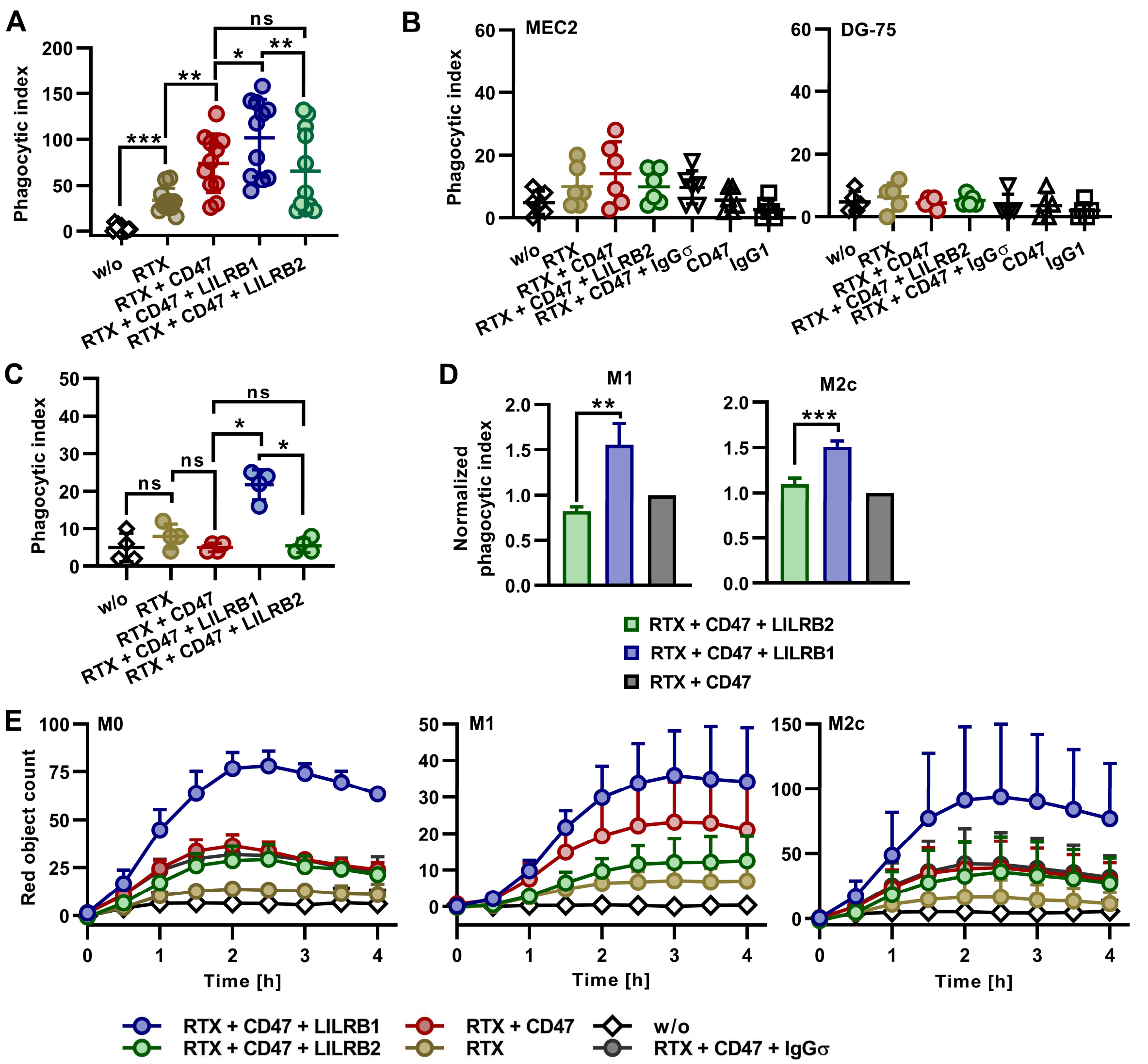

Supplementary Figure 4: LIRB2-IgGσ does not promote ADCP of lymphoma cells by macrophages. (A) LILRB1-IgGσ (LILRB1) or LILRB2-IgGσ (LILRB2) were combined with rituximab (RTX) and CD47-IgGσ (CD47) in 2 h ADCP assays with CSFE-labeled Carnaval cells and M0 macrophages (E:T cell ratio: 1:2). Phagocytic index values were determined by fluorescence microscopy. Antibodies were analyzed at a concentration of 10 µg/ml. Data points represent results from individual experiments with macrophages from different donors. Horizontal lines indicate mean values ± SD (w/o, without added antibody; *, $P \leq 0.05$; **, $P \leq 0.01$; ***, $P \leq 0.001$; ns, not significant; one-way ANOVA with Šidàk´s post-hoc test; $n = 11$). **(B)** ADCP of CFSE-labeled MEC2 ($n = 6$) or DG-75 ($n = 5$) lymphoma cells was analyzed with M0 macrophages without addition of an antibody (w/o) or with antibodies RTX, CD47-IgGσ, LILRB2-IgGσ, HER2-IgGσ (IgGσ) or trastuzumab (IgG1; each at a concentration of 10 µg/ml) by fluorescence microscopy. **(C)** LILRB1-IgGσ and LILRB2-IgGσ were each combined with RTX and CD47-IgGσ in 2 h ADCP assays with DG-75 cells and M0 macrophages. Antibodies were analyzed at a concentration of 10 µg/ml (*, $P \leq 0.05$; ns, not significant; one-way ANOVA with Šidàk´s post-hoc test; $n = 4$). **(D)** M1 and M2c macrophages were incubated with CFSE-labeled Carnaval cells and the antibodies RTX, CD47-IgGσ (CD47), LILRB2-IgGσ (LILRB2) and LILRB1-IgGσ (LILRB1) as assigned, each applied at a concentration of 10 µg/ml. Mean phagocytic index values by antibody triple combinations were normalized to ADCP induced by RTX + CD47-IgGσ. Bars indicate mean values ± SD (**, $P \leq 0.01$, ***, $P \leq 0.001$; one-way ANOVA with Šidàk´s post-hoc test. **(E)** DG-75 lymphoma cells were labeled with pHrodo® and analyzed as target cells for M0, M1 or M2c macrophages in the absence (w/o) or in the presence of RTX alone or in combination with IgGσ antibodies against CD47, LILRB1, LILRB2 or HER2 (IgGσ) as indicated (concentration: 10 µg/ml). Data points represent mean values of red object count per image ± SD ($n = 3$). The E:T cell ratio was 1:2.

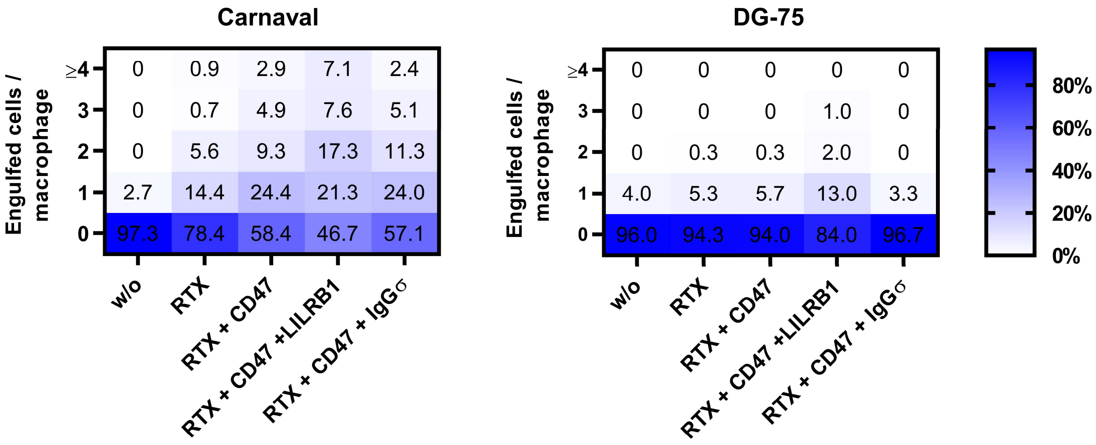

Supplementary Figure 5: Single and multiple ADCP of Carnaval and DG-75 cells. CFSE-labeled Carnaval (*n* = *9*) or DG-75 cells (*n* = 6) were incubated with M0 macrophages in the absence (w/o) or in the presence of antibodies rituximab (RTX), CD47-IgGσ (CD47), LILRB1-IgGσ (LILRB1) or HER2-IgGσ (IgGσ) as indicated (antibody concentration: 10 µg/ml). After 2 h, the number of engulfed lymphoma cells by individual macrophages was determined. Heat maps indicate the percentage of macrophages grouped according to numbers of engulfed lymphoma cells.

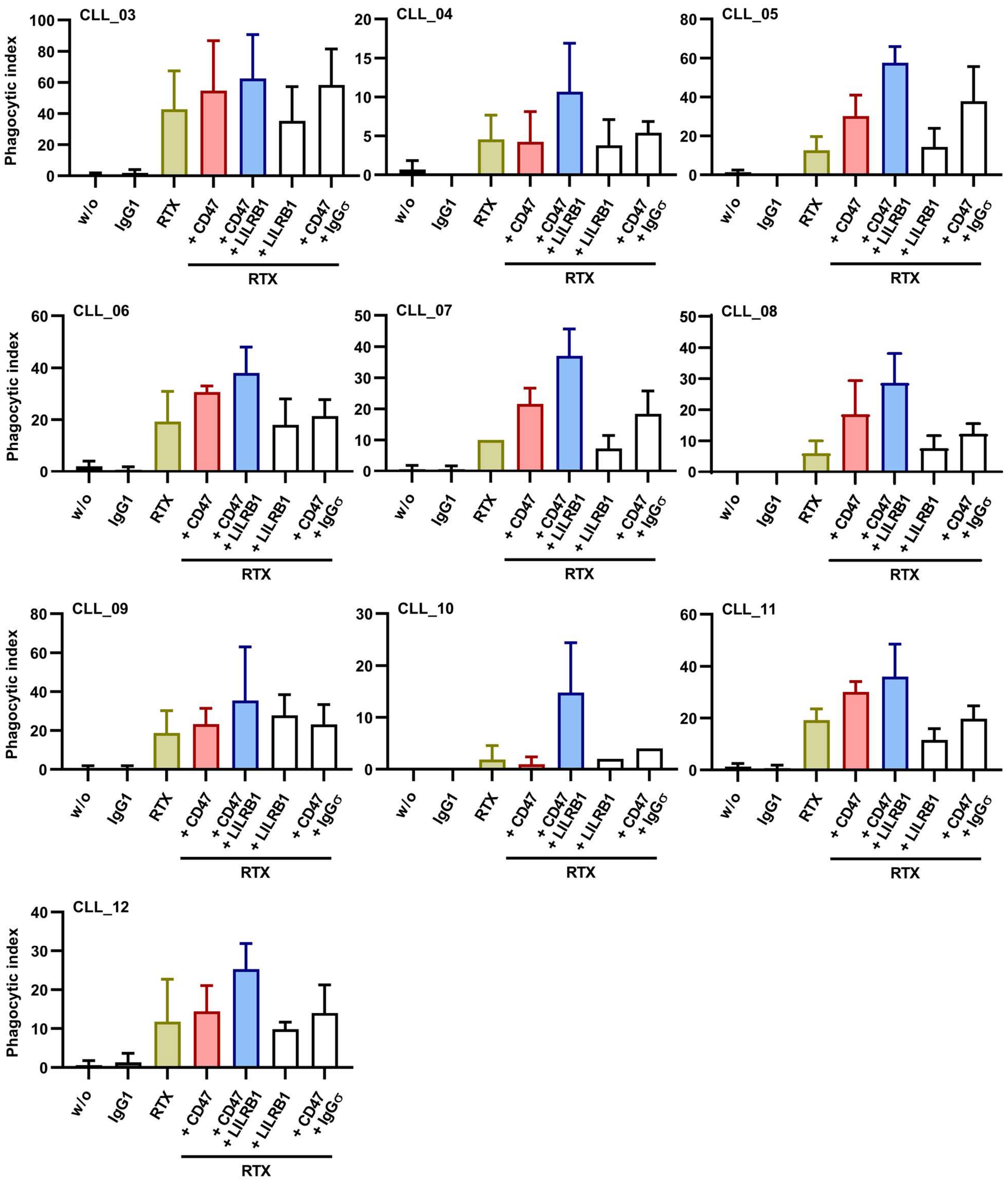

Supplementary Figure 6: ADCP of patient CLL cells. CFSE-labeled CLL cells isolated from the peripheral blood of different patients were incubated with M0 macrophages (E:T cell ratio: 1:2) in the absence (w/o) or in the presence of antibodies trastuzumab (IgG1), rituximab (RTX), LILRB1-IgGσ (LILRB1), CD47-IgGσ (CD47) or HER2-IgGσ (IgGσ) as indicated. Each antibody was analyzed at a concentration of 10 µg/ml. After 2 h, ADCP was determined by fluorescence microscopy. Data points represent mean phagocytic index values ± SD from independent experiments using macrophages from two (CLL_10) or three different donors (all other samples).
